# Homologous recombination suppresses transgenerational DNA end resection and chromosomal instability in fission yeast

**DOI:** 10.1093/nar/gkad160

**Published:** 2023-03-23

**Authors:** Chen-Chun Pai, Samuel C Durley, Wei-Chen Cheng, Nien-Yi Chiang, Jennifer Peters, Torben Kasparek, Elizabeth Blaikley, Boon-Yu Wee, Carol Walker, Stephen E Kearsey, Francesca Buffa, Johanne M Murray, Timothy C Humphrey

**Affiliations:** MRC Oxford Institute for Radiation Oncology & Biology, Department of Oncology, University of Oxford, Old Road Campus Research Building, Roosevelt Drive, Oxford OX3 7DQ, UK; MRC Oxford Institute for Radiation Oncology & Biology, Department of Oncology, University of Oxford, Old Road Campus Research Building, Roosevelt Drive, Oxford OX3 7DQ, UK; MRC Oxford Institute for Radiation Oncology & Biology, Department of Oncology, University of Oxford, Old Road Campus Research Building, Roosevelt Drive, Oxford OX3 7DQ, UK; MRC Oxford Institute for Radiation Oncology & Biology, Department of Oncology, University of Oxford, Old Road Campus Research Building, Roosevelt Drive, Oxford OX3 7DQ, UK; MRC Oxford Institute for Radiation Oncology & Biology, Department of Oncology, University of Oxford, Old Road Campus Research Building, Roosevelt Drive, Oxford OX3 7DQ, UK; MRC Oxford Institute for Radiation Oncology & Biology, Department of Oncology, University of Oxford, Old Road Campus Research Building, Roosevelt Drive, Oxford OX3 7DQ, UK; MRC Oxford Institute for Radiation Oncology & Biology, Department of Oncology, University of Oxford, Old Road Campus Research Building, Roosevelt Drive, Oxford OX3 7DQ, UK; MRC Oxford Institute for Radiation Oncology & Biology, Department of Oncology, University of Oxford, Old Road Campus Research Building, Roosevelt Drive, Oxford OX3 7DQ, UK; MRC Oxford Institute for Radiation Oncology & Biology, Department of Oncology, University of Oxford, Old Road Campus Research Building, Roosevelt Drive, Oxford OX3 7DQ, UK; Department of Biology, University of Oxford, Zoology Research and Administration Building, Mansfield Road, Oxford OX1 3SZ, UK; MRC Oxford Institute for Radiation Oncology & Biology, Department of Oncology, University of Oxford, Old Road Campus Research Building, Roosevelt Drive, Oxford OX3 7DQ, UK; Genome Damage and Stability Centre, School of Life Sciences, University of Sussex, Falmer, Brighton, SussexBN1 9RQ, UK; MRC Oxford Institute for Radiation Oncology & Biology, Department of Oncology, University of Oxford, Old Road Campus Research Building, Roosevelt Drive, Oxford OX3 7DQ, UK

## Abstract

Chromosomal instability (CIN) drives cell-to-cell heterogeneity, and the development of genetic diseases, including cancer. Impaired homologous recombination (HR) has been implicated as a major driver of CIN, however, the underlying mechanism remains unclear. Using a fission yeast model system, we establish a common role for HR genes in suppressing DNA double-strand break (DSB)-induced CIN. Further, we show that an unrepaired single-ended DSB arising from failed HR repair or telomere loss is a potent driver of widespread CIN. Inherited chromosomes carrying a single-ended DSB are subject to cycles of DNA replication and extensive end-processing across successive cell divisions. These cycles are enabled by Cullin 3-mediated Chk1 loss and checkpoint adaptation. Subsequent propagation of unstable chromosomes carrying a single-ended DSB continues until transgenerational end-resection leads to fold-back inversion of single-stranded centromeric repeats and to stable chromosomal rearrangements, typically isochromosomes, or to chromosomal loss. These findings reveal a mechanism by which HR genes suppress CIN and how DNA breaks that persist through mitotic divisions propagate cell-to-cell heterogeneity in the resultant progeny.

## INTRODUCTION

Chromosomal instability (CIN) increases the rate of numerical and structural chromosomal aberrations and is a hallmark of cancer (1). Studies over the last century using cytogenetics, live-cell imaging and, more recently, whole genome sequencing (WGS) have revealed large scale inter- and intratumoural chromosomal abnormalities (2,3). Such heterogeneity has been proposed as a key factor contributing to the lethal outcome of cancer, therapeutic failure and drug resistance (4). Numerical CIN, in which the frequency of gain or loss of whole chromosomes is increased, reflects the loss of chromosome segregation fidelity in mitosis. Such increased chromosomal missegregation can result from distinct mechanisms including defects in chromosome cohesion, mitotic checkpoint function and centrosome copy number (5). Structural CIN refers to the increased frequency of large deletions, amplifications, translocations or other acquired chromosomal aberrations. The increased rate of such events associated with structural CIN appears to be driven through inappropriate mitotic segregation of broken, misrepaired or incompletely replicated chromosomes (6). However, the underlying molecular events that drive structural CIN remain poorly understood.

DNA double-strand break misrepair is a potential driver of CIN and cancer predisposition as highlighted by mutations in BRCA1, ATM, NBS1 and BLM being causal in cancer syndromes associated with CIN (7). DSBs are repaired by either canonical non-homologous end joining (C-NHEJ), homologous recombination (HR) or their alternative pathways, alternative end joining (Alt-EJ) or single-strand annealing (SSA), respectively (8). Importantly, impaired HR has recently been implicated as a major driver of CIN in a wide range of human cancer types (9,10). Genomic analysis has linked somatic mutations in HR genes with significantly elevated levels of genomic CIN signatures, including loss of heterozygosity (LOH), copy number aberrations and tandem duplications. Such signatures are frequently found in breast cancers and ovarian cancers with mutations in or defective expression of BRCA1 or BRCA2 (11). Defective HR could potentially lead to CIN through C-NHEJ or alternative end-joining (A-NHEJ) of DSBs leading to dicentric chromosomal fusions (12). Such dicentrics facilitate CIN through breakage fusion bridge cycles (13). Alternatively, HR defects may promote CIN by generating chromosomal bridges resulting from aberrant processing of one-ended DSBs arising from replication defects or through unresolved HR intermediates (14–16). A distinguishing characteristic of HR repair is the enzymatic processing of the 5’ DNA end to reveal a single-stranded 3’ terminal sequence. Such DSB resection is initiated by removal of a short 5’ DNA tract that requires the MRN complex and CtIP, while extensive resection requires BLM^Hs^ (Sgs1^Sc^, Rqh1^Sp^), EXO1 and DNA2 (17–19). Resected DSB ends subsequently undergo RAD51-dependent homologous strand invasion and DNA replication, thereby facilitating templated DNA repair (18). However, if unrestrained, as a result of failed HR, extensive resection can be genotoxic, leading to chromosomal rearrangements (20,21). Yet, whether extensive DSB end resection contributes to CIN is currently unknown.

DNA damage checkpoints arrest cell division in the presence of DNA damage, thereby preventing the mitotic segregation and propagation of damaged chromosomes (22). However, chromosomes exhibiting persistent damage, arising from unrepaired breaks or telomere loss can be inherited in progeny cells, as a result of checkpoint adaptation (23–26). In budding yeast, checkpoint adaptation can be facilitated by deactivating the DNA damage signal initiated by single-stranded DNA (ssDNA) by impeding 5’ end resection resulting from a DSB (25); through binding ssDNA by combinations of the RPA complex and Rdh54 (Tid1), or Rad52 and Rad51 (27,28); through dephosphorylation of the checkpoint effector kinase Rad53 by inhibiting its phosphorylation by Cdc5 (24,29–32), or through Rad53 dephosphorylation by the Ppc2 phosphatases Ptc2 and Ptc3 (33,34). In mammalian cells, checkpoint adaptation is dependent on Plk1 and correlates with Chk1 dephosphorylation (26,35). Checkpoint adaptation contributes to genomic instability (36–39), and occurs in cancer cells in response to genotoxic chemotherapeutic agents (40,41). As checkpoint adaptation facilitates segregation of broken chromosomes this suggests a mechanism by which CIN may be perpetuated.

While structural CIN leads to the perpetuation of heterogenous chromosomal rearrangements the mechanisms by which such events arise at an increased rate remains poorly understood. In this study we have explored the mechanistic relationship between DSB misrepair and CIN using fission yeast as a model system. From a genome-wide screen of DNA damage-sensitive mutants we establish a common role for HR genes in suppressing DSB-induced CIN. Further, we have followed the fate of an unrepaired broken chromosome across multiple generations, using a combination of physical, imaging, molecular, genetic, and whole genome sequencing approaches. Our findings reveal a series of steps by which, in the absence of HR repair, a broken chromosome carrying a single-ended DSB can propagate widespread CIN. These findings provide new mechanistic insights into how impaired HR drives CIN.

## MATERIALS AND METHODS

### Yeast strains, media and genetic methods

Standard fission yeast media and growth conditions were used throughout this work. Cultures were grown in rich media (YE6S) or Edinburgh minimal media (EMM) at 32°C with shaking, unless otherwise stated (42). See Supplementary Table S6 for strain details.

### DNA damage sensitivity assays

Deletion strains from the Bioneer *S. pombe* haploid deletion library were screened for sensitivity to MMS (0.005%), bleomycin (0.001%) and/or CPT (10 μM) in a 96 well-array format on solid YE6S medium plates and subsequently replica-plated onto the respective drug plates in quadruple spots in duplicate. Sensitive mutants were identified by reduced or absent colony size on the drug plates after 48 hours of incubation at 32°C and confirmed by spot dilution assay.

### The DSB-induced sectoring assay

The sectoring assay is used to detect CIN through an increased rate of loss of a non-essential minichromosome, Ch^16^. Ch^16^ encodes an *ade6-m216* heteroallele, which when lost results in an Ade^−^ cell, leading to pink sectored or striated colonies when grown on low adenine plates (43), thus visualising CIN. G418^R^ strains from the Bioneer V2-deletion library carrying the non-essential minichromosome Ch^16^-*LMYAU* (*leu2, MATa, hygB, ade-m216, ura4*) were generated. Ch^16^-*LMYAU* carries an endogenous copy of the HO-endonuclease gene under an *nmt1* (no message in thiamine) promoter that is induced without thiamine and generates a DSB uniquely at the *MATa* site. Following growth on selective media, containing 2 μM of thiamine (T), cells were plated onto sectoring plates with media containing EMM + LRHUA (low adenine; 7.5mg/L) +/- T to identify break-dependent, and independent Ch^16^ loss; or EMM + RHUA (low adenine) +/– T to identify break-dependent, and independent LOH. Plates were incubated at 32°C for 2–3 days, and subsequently kept at 4°C for 2 days prior to analysis. Sectoring strains were confirmed by repeating the assay a further 2 times.

### DNA double-strand break assays

DSB assays using strains carrying the minichromosomes Ch^16^-DSB (Ch^16^-RMYAH), or Ch^16^-DSB-Tel (Ch^16^-UA-Ch1MGTASTEL) were performed as outlined previously (44). Following DSB induction by HO endonuclease at the *MATa* site in Ch^16^ RMYAH the percentage of colonies undergoing NHEJ/ SCC (Arg^+^ Hyg^R^ Ade^+^ His*^+^*), gene conversion (Arg^+^ Hyg^S^ Ade^+^*His^+^*), Ch^16^ loss (Arg^−^ Hyg^S^ Ade^−^ His^−^) or LOH (Arg^+^ Hyg^S^ Ade^−^ His*^−^*) were calculated. To determine the levels of break-induced GC, Ch^16^ loss and LOH, background events at 48h-T were subtracted from break-induced events at 48 h-T in cells expressing HO endonuclease. Following DSB induction in Ch^16^-DSB-Tel the percentage of colonies undergoing NHEJ/SCC (Ura^+^ Ade^+^ G418^R^), isochromosome (Ch^I^) formation (Ura^+^ Ade^−^ G418^S^), dnTA (Ura^+^ Ade^+^ G418^S^) or Ch^16^ loss (Ura^−^ Ade^−^ G418^S^) were calculated as described above. More than 1000 colonies were scored for each time point. Mean ± SEM values were obtained from triplicate experiments. Differences were deemed significant if *p*-values obtained using Student's *t* test were ≤ 0.05.

### Pulse field gel electrophoresis

The procedures used in this study for PFGE analysis have been described previously (45). For the time course experiment, Ch^16^-DSB-Tel cells were inoculated in EMM + U + A + L + R medium (+T or –T). Samples were collected and washed twice in 0.05 M EDTA at indicated time points before PFGE analysis.

### Pedigree analysis

The procedures used in this study for pedigree analysis have been described previously (46).

### Protein analysis

Proteins were extracted using trichloroacetic acid (TCA) precipitation and analysed by western blotting as described previously (47). TAP-tagged proteins were detected with peroxidase–anti-peroxidase–soluble complex (P1291, Sigma). α-tubulin was detected with antibody T5168 (Sigma).

### Microscopy analysis

Ch^16^-DSB-Tel carrying cells were inoculated in EMM medium in the presence or absence of thiamine at 32°C. Samples were collected at indicated time points, fixed in methanol/acetone, rehydrated and stained with 4′,6-diamidino-2-phenylindole (DAPI) before examination using Zeiss Axioplan 2ie microscope, Hamamatsu Orca ER camera. Open-source micromanager software was used to analyse the images (48).

### Live cell imaging

Live cell imaging was performed on agarose pads as previously described (49). Imaging was performed on a Nikon Ni-E inverted microscope at 100× magnification in a temperature-controlled environment chamber at 32°C. Multiple XY positions were taken, and two-channel images (transmitted light and GFP) were taken over 5 Z-stacks of 0.4 μm. Imaging was performed every 20 min for up to 18 h. Deconvolution of the GFP channel was performed using Nikon's NIS-Elements software using an automatic algorithm. The images shown are maximum projections of the deconvolved image GFP-channel and a single-plane image from the transmitted light channel.

### Whole genome DNA sequencing

*S. pombe* DNA was extracted from cells grown to log phase at 32°C using MasterPureTM Yeast DNA purification kit (Lucigen). Genomic DNA from strains carrying Ch^16^-DSB-Tel, and loss of heterozygosity (LOH) strains LOH1, LOH5 and LOH9 was sent to Novogen (UK) Company Limited for whole genome sequencing (Illumina PE150). The resulting reads were aligned to the reference genome *Schizosaccharomyces pombe* ASM294v2 using bowtie2 v2.2.6. Only the locations with best alignment scores were kept if the reads aligned to multiple locations. The average coverage rate over all samples and all chromosomes is 64.7 reads. The number of reads over equally sized regions were counted by python package HTSeq. The sequence data are available using the link: https://dataview.ncbi.nlm.nih.gov/object/PRJNA795979?reviewer=op9hm1s1oocds7lod9aga90ikq

## RESULTS

### HR suppresses break-induced chromosomal instability

**Figure 1.**
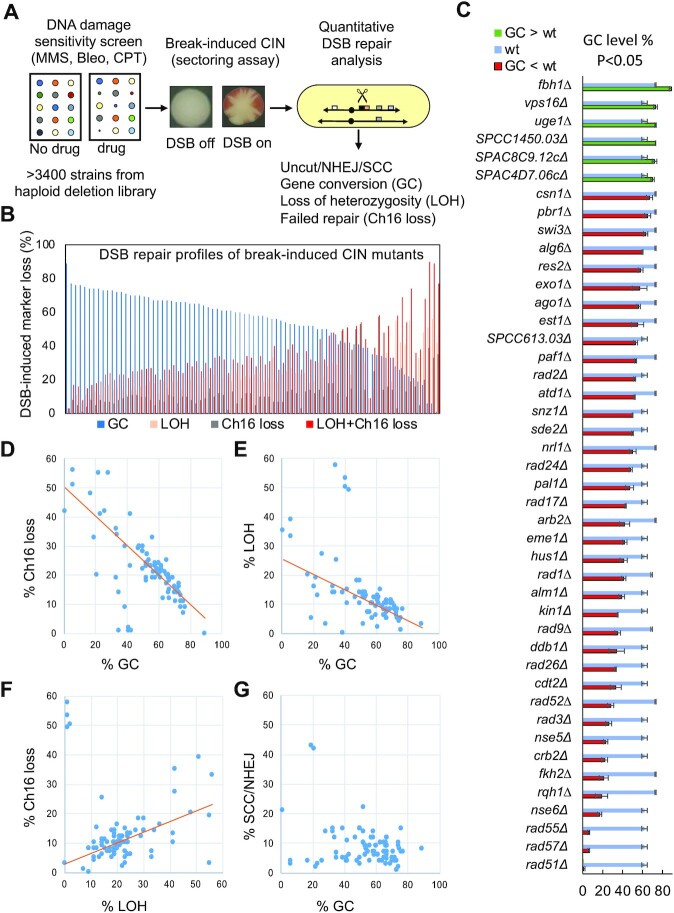
HR genes suppress break-induced CIN. (**A**) Identification of break-induced CIN mutants. Bioneer *S. pombe* haploid deletion libraries were screened for deletion strains with sensitivity to MMS (0.005%), bleomycin (0.001%) or CPT (10 μM) as indicated in Materials and Methods. Sensitive strains are indicated in Supplementary Table S1. Strains with a reduced colony size on plates with DNA damaging agents compared to wild type were further subjected to a break-induced sectoring assay utilising a non-essential minichromosome Ch^16^-LMYAU to indicate an increased rate of CIN (see Materials and Methods). A quantitative DSB repair analysis was subsequently performed on mutants identified in the break-induced sectoring assay. Details of the quantitative DSB assay are described in Materials and Methods and Supplementary Figure S1. For further experimental details see (44); For strain details see Supplementary Table S6. (**B**) DSB repair profiles of break-induced CIN mutants. Percentage break-induced GC (blue) is plotted against percentage break-induced LOH (orange), Ch^16^ loss (grey), and LOH + Ch^16^ loss (red) for each of the mutants analysed (Supplementary Table S3). For each genetic background the DSB assay was repeated three times, to score > 600 colonies. Mean ± the standard deviation of the 3 experiments are shown. A single blank vector control was also analysed in each genetic background to give a spontaneous level of Ch^16^ loss that was subtracted to calculate the break-induced values shown above. Ch^16^-RMYAH was used here instead of Ch^16^-LMYAU due to marker selection requirements in the experiment procedure. (**C**) Plot of individual CIN mutants exhibiting significantly altered gene conversion (GC) levels compared to wild type. CIN mutants identified in screen (A) exhibiting significantly reduced (red bars) or elevated (green bars) GC levels compared to their respective wild-type background (Ch^16^-RMYAH) performed under the same experimental conditions (blue bars; P ≤ 0.05) are indicated (Materials and Methods; Supplementary Table S3). (**D**) Percentage break-induced GC is plotted against percentage break-induced Ch^16^ loss for each of the mutants analysed (regression line: *y* = –0.5061*x* + 50.438, *R*² = 0.6863). (Supplementary Table S3). (**E**) Percentage break-induced GC is plotted against percentage break-induced LOH for each of the mutants analysed (*y* = –0.2631*x* + 25.478, *R*² = 0.4984). (Supplementary Table S3). (**F**) Percentage break-induced Ch^16^ loss is plotted against percentage break-induced LOH for each of the mutants analysed (*y* = 0.362*x* + 2.8594, *R*² = 0.3523). (Supplementary Table S3). (**G**) Percentage break-induced SSC/NHEJ is plotted against percentage break-induced GC for each of the mutants analysed (Supplementary Table S3).

To explore the relationship between DSB misrepair and CIN we performed an unbiased genome-wide screen to identify DSB repair mutants exhibiting elevated levels of break-induced CIN. As a first step we screened a library of haploid deletion mutants (>3400 strains; (50)) to identify mutants sensitive to DNA damage by the radiomimetic bleomycin, the alkylating agent methyl methanesulfonate (MMS) or the topoisomerase inhibitor, camptothecin (CPT) (Figure 1A; Materials and Methods). A total of 359 mutants were confirmed as sensitive to at least one of these DNA damaging agents (Supplementary Table S1), yielding a comprehensive list of deletion mutants conferring sensitivity to specific types of DNA damage, with 54 mutants sensitive to all three of these DNA damaging agents.

To explore the potential role of these DNA repair mutants in CIN, we examined the impact of their deletion on DSB-induced sectoring. To do this, we used a strain carrying a stable non-essential 530 kb minichromosome (Ch^16^), experimentally derived from the centromeric region of chromosome III (ChIII) (51). Ch^16^ was adapted such that, following HO endonuclease derepression, a DSB is induced at a *MATa* site within Ch^16^ (Ch^16^-LMYAU) (52). Using this approach, increased rates of either Ch^16^ loss or chromosomal rearrangements can result in loss of the *ade6-m216* heteroallele and an Ade^-^ phenotype that can be detected as pink sectoring colonies, indicative of CIN (Figure 1A; Materials and Methods). We found that 95 out of 359 mutants exhibited break-induced colony sectoring, and thus CIN. This revealed a set of break-induced CIN genes whose gene ontology (GO) terms include DNA repair, checkpoint, chromatin remodelling, transcription, RNA interference, protein transport, ubiquitylation and metabolism (Supplementary Table S2).

We wished to determine the DSB repair/misrepair profiles of these CIN mutants. Using a DSB assay (Figure 1A; Supplementary Figure S1), it is possible to quantitate levels of DSB repair, misrepair and failed repair events in most genetic backgrounds (44). We have previously shown that a DSB induced within Ch^16^ is efficiently repaired by HR, the major DSB repair pathway in fission yeast, resulting in gene conversion (GC) of a marker adjacent to the DSB site, using the homologous ChIII as a repair template (53). In contrast, failed HR repair leads to minichromosome loss (Ch^16^ loss) or to extensive loss of heterozygosity (LOH), resulting in differential loss of distant genetic markers integrated within Ch^16^ through either isochromosome formation (21) or *de novo* telomere addition (54) (Supplementary Figure S1). 81 sectoring mutants were successfully crossed with the minichromosome strain Ch^16^-RMYAH (termed here Ch^16^–DSB), and DSB repair outcomes analysed. The majority (66/81) of the CIN mutants exhibited a significantly altered DSB repair profile compared to a wild-type background, including significantly altered levels of sister chromatid conversion (SCC), interchromosomal gene conversion (GC), break-induced Ch^16^ loss, or LOH (Figure 1B; Supplementary Table S3; Supplementary Figure S2). Of these, 44 CIN genes exhibited significantly altered interchromosomal GC levels (P ≤ 0.05), with 38 mutants exhibiting reduced (Figure 1C, red bars) and 6 with increased GC levels (Figure 1C, green bars) compared to a wild-type background (Figure 1C, blue bars), indicative of dysfunctional HR repair (Supplementary Table S3). This group includes many previously characterized HR genes such as, *rad51^+^*, *rad52^+^*, *rad55^+^*, *rad57^+^*, *rqh1^+^* (BLM^Hs^) and *fbh1^+^*; genes impacting on HR, including *ddb1^+^, cdt2^+^*; DDR factors, *rad3^+^* (ATR), *hus1^+^*, *rad9^+^*, *rad17^+^*, *rad26^+^*, (52,55,56), as well as newly described genes impacting on HR such as *ago1^+^, alm1^+^, snz1^+^, kin1^+^, pal1^+^* and *paf1^+^*; the majority of which are evolutionarily conserved (Figure 1C and Supplementary Table S3). Analysis of DSB repair profiles across all these mutants revealed a gradient of GC levels (Figure 1C). This suggests that some genes, for example *rad51^+^*, are more intimately involved in HR repair than others, although weaker HR genes may perform redundant functions. Moreover, a striking inverse correlation between GC levels and failed repair events (LOH + Ch^16^ loss) was observed (Figure 1B). Accordingly, linear regression analysis revealed GC levels to correlate inversely with Ch^16^ loss (Figure 1D) and with LOH (Figure 1E), while LOH positively correlated with Ch^16^ loss (Figure 1F). In contrast, the majority of mutants exhibited similar levels of SCC/NHEJ to wild-type cells irrespective of the GC levels (Figure 1G). These results indicate that failed GC does not lead to increased NHEJ but instead gives rise to elevated levels of both Ch^16^ loss and extensive LOH. These findings together identify a common role for HR genes in suppressing numerical and structural CIN. Importantly, orthologues of 17 out of these 44 HR genes have been previously identified as CIN genes in *S. cerevisiae* (57) (Supplementary Table S4), suggesting that break-induced CIN in HR mutants may arise through a common evolutionarily conserved mechanism.

**Figure 2.**
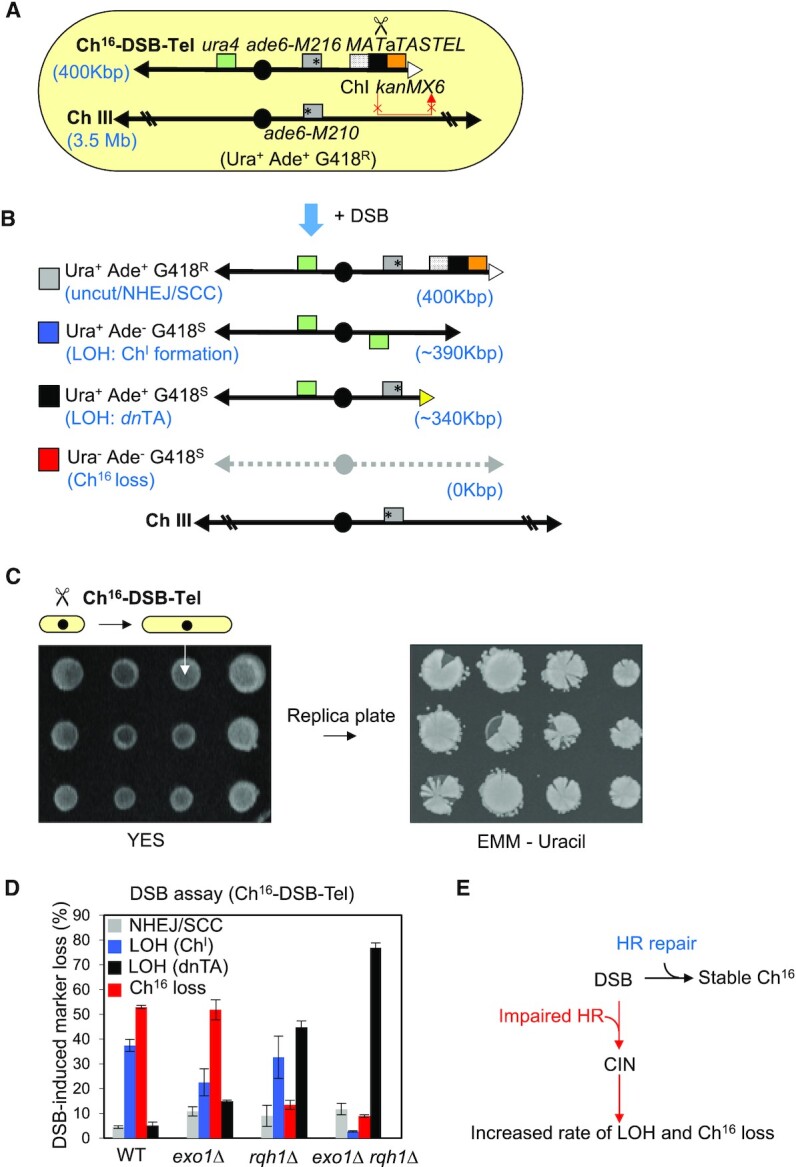
Structurally disrupting HR repair of a DSB drives CIN. (**A**) Schematic of minichromosome Ch^16^‐Tel-DSB and homologous ChIII. The *ura4* gene was integrated into the *mad3^+^* locus (green box), of Ch^16^‐Ch1MGTastel (Supplementary Figure S3) to form Ch^16^‐Tel-DSB. The loci of natural telomeres (black triangles), the artificial telomeric sequence (TASTEL) (white triangle), centromere (black circles), complementary *ade6-M216* and *ade6-M210* heteroalleles (grey boxes with asterisk denoting point mutations), 3 kb region of homology to ChI (black dots), *MAT*a target site (black box), and *KanMX6* gene (G418^R^) (salmon), are indicated. (**B**) Schematic of potential repair and misrepair outcomes following HO-induced DSB at *MATa* site within Ch^16^-DSB-Tel (A). Derepression of *nmt141x-HO* integrated into *ARS1* in the absence of thiamine (–T) generates a DSB uniquely at the *MAT*a target site (scissors). Repair of an HO-induced DSB by HR typically uses the homologous ChIII as a template (red arrows). However, gene conversion is not possible following DSB induction at the *MATa* site in Ch^16^-DSB-Tel due to the absence of the homologous distal Ch^16^ arm, thus abrogating second-end capture and therefore HR repair, and the presence of 3 kb non-homologous ChI sequence (see Supplementary Figure S3). Repair of HO-induced DSB by NHEJ or sister chromatid conversion (SCC) if only one sister chromatid is broken results in retention of all markers resulting in an Ura^+^ Ade^+^ G418^R^ phenotype, which is indistinguishable from the uncut minichromosome. Extensive LOH, in which genetic material centromere-distal to the break-site is lost, results in Ura^+^ Ade^−^ G418^S^ phenotype, and arises predominantly from isochromosome (Ch^I^) formation, as indicated. Extensive LOH, resulting in Ura^+^ Ade^+^ G418^S^ phenotypes, usually results from *de novo* telomere addition (*dn*TA) occuring between the *ade6-M216* allele and the *MATa* break site (yellow triangle). Failed DSB repair can also result in loss of the minichromosome (Ch^16^ loss), resulting in a Ura^−^ Ade^−^ G418^S^ phenotype. See Materials and Methods. (**C**) Sectoring analysis of colonies formed from individual elongated Ch^16^-DSB-Tel cells grown on YES plates following DSB induction. Unselected colonies were replica plated onto uracil- plates and incubated at 32°C for 2–3 days. (**D**) Percentage of DSB-induced marker loss in wild type, *exo1Δ* (TH6671), *rqh1Δ* (TH7532), or *rqh1Δ exo1Δ* (TH7536) backgrounds carrying Ch^16^‐Tel-DSB. The levels of uncut/NHEJ/SCC, Ch^16^ loss and LOH are shown (see also A and B). SEM values are indicated. The data presented are from at least two independent biological repeats. (**E**). HR suppresses DSB induced chromosomal instability in fission yeast. Genetically or structurally impaired HR leads to significantly increased CIN leading to elevated rates of LOH and chromosome loss.

In addition to disrupting HR genetically, we considered whether disrupting HR structurally might also lead to elevated levels of CIN. To achieve this, we replaced the Ch^16^ minichromosome arm distal to the *MATa* break site with an adjacent G418 resistance marker and synthetic telomere, thereby abrogating second-end capture following DSB induction, and instead giving rise to an unrepairable single-ended DSB (Figure 2A; Supplementary Figure 3S, TH2039). To further compromise GC, a 3 kb non-homologous region of ChI was integrated directly proximal to the break site, thus abrogating strand invasion into the homologous ChIII repair template (Figure 2A; Supplementary Figure S3, TH2055). DSB induction in this context is predicted to generate a single-ended DSB which no longer has proximal homology to ChIII, and is further unable to complete second-end capture, and thus HR repair (Figure 2A, red arrow). Instead, DSB induction was predicted to lead to high levels of DSB misrepair events associated with failed HR repair including minichromosome loss (Ch^16^ loss) or to extensive loss of heterozygosity (LOH), resulting in differential loss of distant genetic markers integrated within Ch^16^ through either isochromosome formation (21) or *de novo* telomere addition (54) (Figure 2B). Consistent with this, DSB induction in this context resulted in a strikingly high degree of failed repair (69%; TH2039), with the addition of the 3 kb non-homologous ChI region resulting in a further increase in failed repair (88%; TH2055) (Supplementary Figure S3). To further test sectoring levels in this context a *ura4* marker was introduced on the left arm of Ch^16^-Ch1MGTastel (TH2055; Supplementary Figure S3) to generate Ch^16^-DSB-Tel (Figure 2A). As predicted, DSB induction in Ch^16^-DSB-Tel in a wild-type background resulted in high sectoring levels, when colonies were replica-plated to ura- plates, indicative of high levels of Ch^16^ loss, and thus CIN (Figure 2C). Consistent with this, quantification of DSB-induced marker loss confirmed high levels of Ch^16^ loss and isochromosome formation (Figure 2B, D), thus mimicking genetic disruption of HR (Figure 1, Supplementary Figure S1, Supplementary Table S3). Break-induced replication (BIR) was not observed in this context, with or without the addition of the 3 kb fragment with homology to the subtelomeric region of ChI (Supplementary Figure S3). Together, these findings indicate that disrupting HR either genetically or structurally results in significantly elevated levels of numerical or structural CIN (Figure 2E). Further, the results of DSB induction in the context of Ch^16^-DSB-Tel suggests that CIN arises through generating an unrepaired single-ended DSB.

### Extensive DSB end resection resulting from failed HR repair proceeds over multiple generations

**Figure 3.**
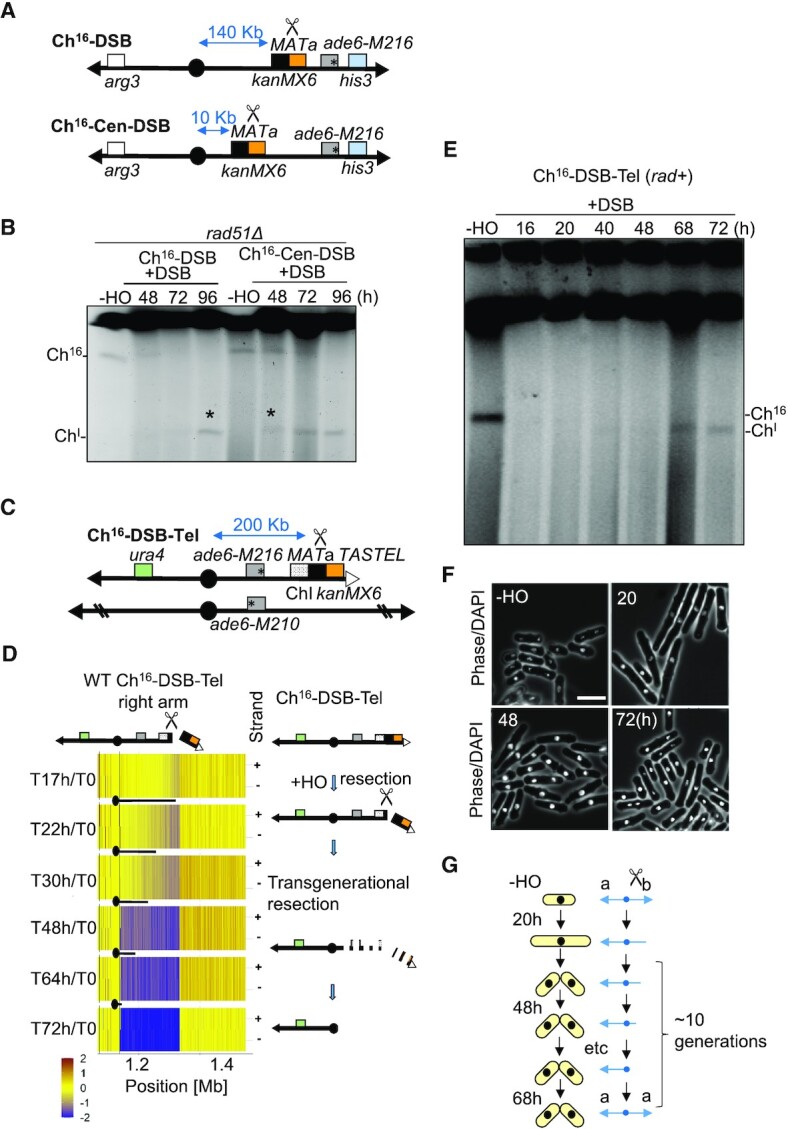
Extensive DSB end resection resulting from failed HR repair proceeds over multiple generations. (**A**) Schematic of Ch^16^-DSB (Ch^16^‐RMGAH) (21), in which the distance from the *MATa* site to the centromere is 140 kb; and Ch^16^-Cen-DSB (Ch^16^-R-*mid1*-MGAH) in which the distance from the *MATa* site to the centromere is 10 kb (*MATa* KanMX6 cassette integrated into the Ch^16^‐DSB *mid1* gene). (**B**) PFGE analysis of samples taken from *rad51Δ* cells carrying Ch^16^‐DSB (TH7356) or Ch^16^‐Cen-DSB (TH6590) grown in absence of thiamine (–T) for the indicated times. Bands corresponding to minichromosome (Ch^16^), and isochromosome (Ch^I^) are indicated. Asterisks indicate isochromosome formation. (**C**) Schematic of Ch^16^-DSB-Tel and ChIII as detailed in Figure 2A. (**D**) Next generation sequence analysis confirms Ch^I^ formation results from sequential loss of the broken chromosome arm. Next generation sequence analysis of Ch^16^‐Tel-DSB cells in a wild-type background following DSB induction with samples taken at times indicated. NGS analysis showing the log_2_ of the signal ratio between the right arm of Ch^16^-DSB-Tel and the right arm of Ch III, as indicated in Figure 2A. An illustration of extensive transgenerational resection is depicted to the right. Data acquisition and normalization were carried out as described in Materials and Methods. Yellow indicates a 1:1 ratio, red indicates signal intensity >1 and blue is <1. (**E**) PFGE analysis of samples taken from Ch^16^-DSB-Tel cells grown in absence of thiamine for the indicated times. Bands corresponding to minichromosome Ch^16^-DSB-Tel (abbreviated to Ch^16^), and isochromosome (Ch^I^) are indicated. (**F**) Cell morphology analysis of Ch^16^-DSB-Tel cells grown in the absence of thiamine for the indicated times. Samples were taken at indicated points in parallel to (**E**) for microscopy analysis. Scale bar = 10 μm. (**G**) Schematic depicting DSB (scissors)-induced Ch^I^ formation occurs over multiple generations in *S. pombe* (yellow cells). DSB induction initially leads to checkpoint-dependent cell cycle delay prior to division. Left (a) and right arm (b) of Ch^16^-DSB-Tel are indicated. Ch^I^ formation is calculated to take up to 10 generations.

We wished to understand the mechanism by which impaired HR repair can lead to CIN. We have previously shown that HR genes suppress isochromosome formation arising from extensive end resection of an unrepaired DSB within Ch^16^. Such extensive resection leads to removal of the broken chromosome arm and to replication of the intact arm from the centromere, hundreds of kilobases from the initial lesion (21). Importantly, in the absence of HR, such isochromosome formation was associated with break-induced colony sectoring, suggesting that this chromosomal rearrangement was a consequence of CIN and possibly occurring over several generations. To test this hypothesis, we first sought to determine the kinetics of isochromosome formation in the absence of HR, and whether it was dependent on the resection distance from the *MATa* break site to the centromere inverted repeats. We therefore performed a time course to monitor isochromosome formation following induction a DSB at a *MATa* site inserted within the body of Ch^16^–DSB (Ch^16^-RMGAH) in a *rad51Δ* background (Figure 3A). We found that efficient isochromosome formation was maximally observed at 96 h following HO endonuclease derepression as previously described (Figure 3B) (21). Next, we moved the *MATa* break site from 140 kb to 10 kb away from the centromere (Ch^16^-Cen-DSB) and found isochromosome formation was observed much earlier (48h) (Figure 3A, B). This suggested that the timing of isochromosome formation is proportional to the resection distance from the break site to the centromeric inverted repeats. We next examined isochromosome formation following DSB induction when HR was structurally impaired, using cells carrying Ch^16^-DSB-Tel. Isochromosome formation was significantly reduced following deletion of both Rqh1 (BLM^Hs^, Sgs1^Sc^) and Exo1 in *rqh1Δ exo1Δ* Ch^16^-DSB-Tel cells, consistent with isochromosome formation requiring extensive DNA end resection (19,21) (Figure 2D). Instead, DSB induction in an *rqh1Δ exo1Δ* background resulted in significantly elevated levels of genetic marker loss consistent with *de novo* telomere addition (*dn*TA) at or near the DSB (Figure 2D), as previously described (54). Together, these findings indicate that genetically or structurally impaired HR leads to extensive DSB-end resection and predominantly to isochromosome formation.

To physically confirm that isochromosome formation arises from extensive resection from an unrepaired DSB and to monitor its kinetics we performed next generation sequence (NGS) analysis of time course samples following DSB induction. Sequence samples were compared to the parental strain carrying Ch^16^-DSB-Tel (T0) (Figure 3C; Figure 2A). NGS analysis confirmed that isochromosome formation resulted from extensive resection from the DSB site to the centromere of Ch^16^-DSB-Tel cells, resulting in sequential loss of the right arm of Ch^16^-DSB-Tel in a wild-type background (Figure 3D). While the timing of DSB induction was heterogenous the resection step alone was calculated to proceed for at least 31 hours (48h-17h DSB induction time) in wild-type Ch^16^-DSB-Tel cells.

As the length of a normal cell cycle for *S. pombe* is 3.5 h, (42), we considered the possibility that either cells undergo a long DNA damage checkpoint-dependent cell cycle delay to accommodate such extensive processing prior to division, or that such break-induced isochromosome formation proceeds across multiple cell divisions. To determine whether cells continued to divide during this time, a detailed time course was performed following DSB induction in wild-type Ch^16^-DSB-Tel cells, in which both isochromosome formation and cell morphology were monitored in parallel (Figure 3E and F). Microscopy analysis indicated that DNA damage checkpoint-dependent cell cycle elongation was observed 20 h after expression of HO endonuclease (–T) (Figure 3F, Supplementary Figure S4A and B). These cells then returned to their normal length and had resumed cell division by 48 h (Figure 3F). This time point was considerably earlier than the time at which efficient isochromosome formation was first observed (68 h) (Figure 3E). This indicates that isochromosomes are formed over multiple generations. Further, these results together suggest that daughter cells inherit an unrepaired broken chromosome resulting from failed HR, and that isochromosome formation is a consequence of CIN, which results from extensive single-ended DSB processing over multiple generations (Figure 3G).

### Checkpoint adaptation enables transgenerational DNA resection

**Figure 4.**
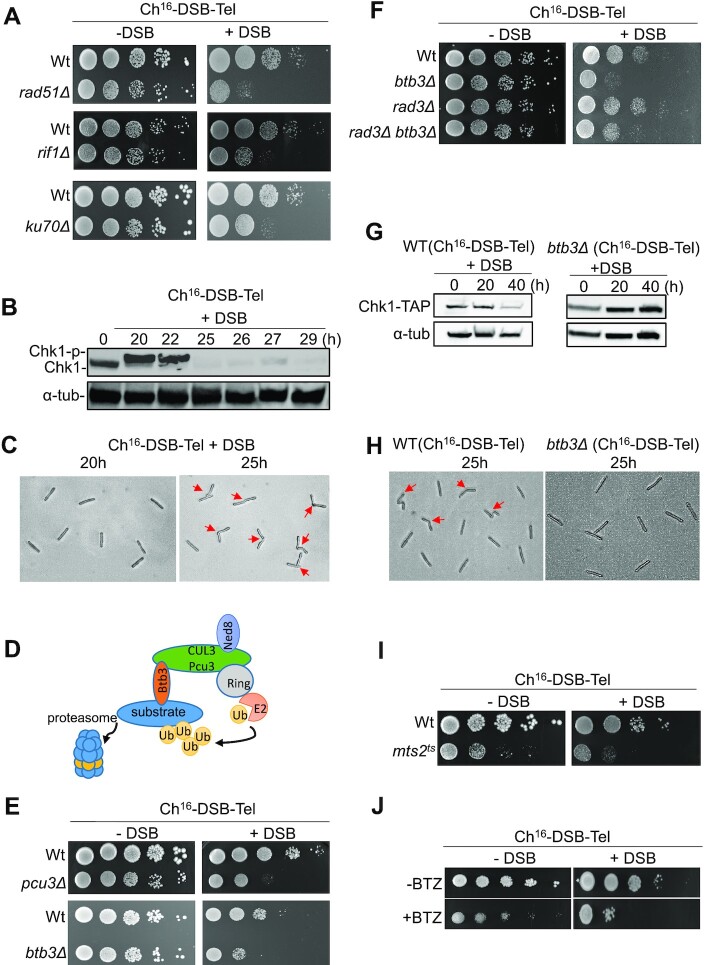
A single-ended DSB facilitates checkpoint adaptation through Chk1 loss. (**A**) Viability analysis of wild-type (TH6864), *rad51*Δ (TH8075), *rif1*Δ (TH9161) or *ku70*Δ cells (TH8276) carrying Ch^16^-DSB-Tel and *nmt1(41X)-HO* integrated into the *ARS1* locus. Cells were serially diluted (10-fold) and spotted onto Edinburgh Minimal Medium (EMM) + U+A + L + R in the presence (-DSB) or absence of thiamine (+DSB) and incubated at 32°C for 2–3 days. (**B**) Analysis of Chk1 protein levels in cells carrying Ch^16^-DSB-Tel following DSB induction at times indicated. Cells were incubated at 32°C and samples taken at the indicated times following thiamine removal (0 h) to derepress *nmt1(41x)-HO* (+DSB). Cell extracts were made by using the TCA method. Tap-tagged Chk1 was detected using an anti-TAP antibody. α-Tubulin is shown as a loading control. (**C**) Microscopy analysis of cells taken at 20 h and 25 h following thiamine removal. Red arrows indicate dividing cells. (**D**) Schematic representation of the Cullin 3-ring ubiquitin ligase (88). (**E**) Viability analysis of wild-type (TH6864), *pcu3*Δ (TH9423) or *btb3*Δ (TH9229), cells carrying Ch^16^-DSB-Tel. Cells were serially diluted (10-fold) and spotted onto EMM plates in the presence (–DSB) or absence of thiamine (+DSB) and incubated at 32°C for 2–3 days. (**F**) Viability analysis of wild-type (TH6864), *btb3*Δ (TH9229), *rad3*Δ (TH9233) or *btb3Δ rad3Δ* (TH9244) cells carrying Ch^16^-DSB-Tel. Cells were serially diluted (10-fold) and spotted onto EMM plates in the presence (–DSB) or absence of thiamine (+DSB) and incubated at 32°C for 2–3 days. (**G**) Analysis of Chk1 protein levels in *btb3*Δ Ch^16^-DSB-Tel cells (TH9229) following DSB induction at times indicated. Cell extracts were made by using the TCA method. Tap-tagged Chk1 was detected using an anti-TAP antibody (upper panel). α-Tubulin is shown as a loading control (lower panel). (**H**) Microscopy analysis of wild-type (TH6864) or *btb3*Δ cells carrying Ch^16^-DSB-Tel (TH9229) taken at 25 h following thiamine removal. Red arrows indicate dividing cells. (**I**) Viability analysis of wild-type (TH6864), and the 19S proteasome mutant *mts2^ts^* cells carrying Ch^16^-DSB-Tel (TH9226). Cells were serially diluted (10-fold) and spotted onto EMM plates in the presence (-DSB) or absence of thiamine (+DSB) and incubated at 32°C for 2–3 days. (**J**) Viability analysis of wild-type cells carrying Ch^16^-DSB-Tel (TH6864) in the presence of proteasome inhibitor Bortezomib (BTZ) following DSB induction. Cells were serially diluted (10-fold) and spotted onto PMG +/– thiamine plates in the presence or absence of Bortezomib (200 μM) and incubated at 26°C for 3 days.

A prediction of the above results is that propagation of an unrepaired broken chromosome over multiple generations requires DNA damage checkpoint adaptation. Checkpoint adaptation has been previously described in fission yeast (58), but is not as well characterized as in *S. cerevisiae* (23–25). To determine if fission yeast carrying the unrepaired broken chromosome were undergoing checkpoint adaptation we tested the impact of deletion of *S. pombe* orthologues of *S. cerevisiae* DNA damage checkpoint adaptation genes to determine their impact on colony formation following DSB induction. We found that deletion of *rad51*, *rif1*, *ku70* and *srs2*, whose orthologues are required for checkpoint adaptation in *S. cerevisiae* (25,28,36,59) resulted in increased cell death following DSB induction in the nonessential minichromosome Ch^16^-DSB-Tel (Figure 4A and Supplementary Figure S4C). In contrast, abrogating the DNA damage checkpoint by deleting *rad3^+^* (ATR^Hs^) (60) did not cause increased viability loss following DSB induction (Supplementary Figure S4C). Consistently, deleting *rad3^+^* leads to elevated isochromosome formation compared to a wild type (Supplementary Figure S4D). These observations strongly suggest that wild-type fission yeast cells divide in the presence of an unrepaired broken chromosome, as a result of checkpoint adaptation, thereby permitting the propagation of an unrepaired broken chromosome.

### Cullin3-mediated Chk1 loss promotes checkpoint adaptation

Chk1, a key effector of the DNA damage checkpoint pathway, is phosphorylated in response to DNA damage (61). To explore the mechanism of checkpoint adaptation, we looked at Chk1 phosphorylation over time following DSB induction. DSB induction in Ch^16^-DSB-Tel cells was associated with increased Chk1 phosphorylation levels at earlier time points when cell division was arrested, consistent with G2-M checkpoint activation (61) (Figure 4B 20–22 h and Figure 4C, 20 h). Unexpectedly, Chk1 protein levels were reduced at 25 h when cells were observed to initiate cell division (Figure 4B, 25–29 h and Figure 4C, 25 h). These results suggest that following initial DNA damage checkpoint activation, Chk1 is lost to facilitate adaptation in response to an unrepaired broken chromosome.

To determine the mechanism of Chk1 loss in more detail, we performed a genome-wide screen for mutants that were unable to adapt following induction of an unrepairable DSB, resulting in DSB-induced synthetic lethality (Supplementary Figure S5). We identified 162 hits (Supplementary Table S5), including deletion of *rad51^+^*, previously found to be required for checkpoint adaptation in *S. cerevisiae* (27,28). In addition, we found that adaptor protein Btb3 of the Cullin3/Pcu3 E3 ubiquitin ligase, not previously implicated in checkpoint adaptation, to be required for cell viability in strains carrying Ch^16^-DSB-Tel following DSB induction (Figure 4E). We explored the possibility that Btb3 E3 ubiquitin ligase may contribute to checkpoint-dependent degradation of Chk1. Consistent with this we found that deletion of *rad3^+^* partially suppressed the lethality of *btb3Δ* Ch^16^-DSB-Tel following DSB induction (Figure 4F), suggesting this pathway is required for Chk1 degradation and checkpoint adaptation in the presence of persistent DNA damage. Further, Western blot analysis showed that Chk1 protein levels remained high in the absence of Btb3 (Figure 4G). Consistent with this notion, loss of Btb3 leads to cell cycle arrest in Ch^16^-DSB-Tel in the presence of a DSB (Figure 4H). Further, Pcu3, another component of the Cullin3 complex, was also found to be required for viability following DSB induction, supporting a role for this complex being required for checkpoint adaptation (Figure 4E). Moreover, inactivation of the fission yeast 19S proteasome component resulted in cell death in the temperature-sensitive mutant *mts2-1* following DSB induction within Ch^16^-DSB-Tel (Figure 4I). Consistently, the proteasome inhibitor Bortezomib (BTZ) modestly increased cell death following DSB induction (Figure 4J). These results suggest that the unrepaired broken chromosome leads to Cullin-3-dependent Chk1 loss and adaptation.

Our data indicate that, once initiated, DNA damage checkpoint adaptation persists over multiple generations. We therefore wished to test the impact of rechallenging cells with DNA damage on cell cycle checkpoint engagement following adaptation. We found that challenging adapted cells following DSB induction (Supplementary Figure S4E) with Bleocin for 3 h resulted in an elongated cell phenotype (Supplementary Figure S4F). These findings indicate that challenging cells that have undergone DNA damage checkpoint adaptation with DNA damage reengages the cell cycle checkpoint machinery resulting in cell cycle delay. These findings resemble those observed in *S. cerevisiae* (38). Together these findings provide new mechanistic insights into how an unrepaired broken chromosome can bypass the DNA damage checkpoint in *S. pombe*.

### Inherited unrepaired broken chromosomes undergo DNA replication

**Figure 5.**
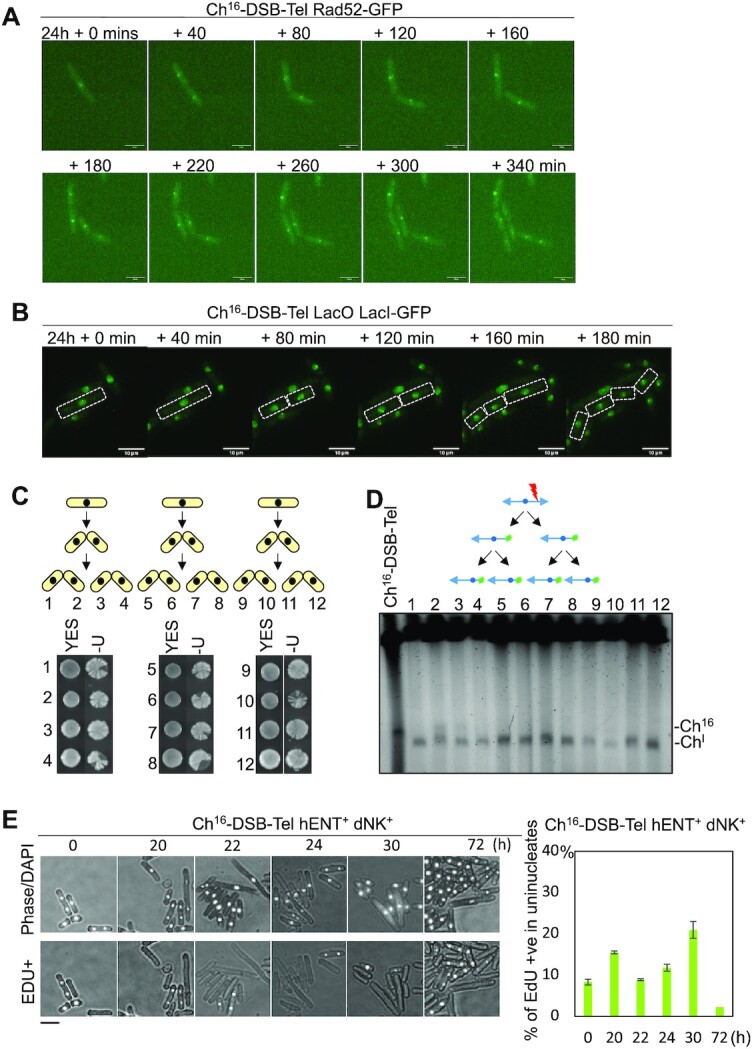
Inherited broken chromosomal elements undergo DNA replication. (**A**) Analysis of Rad52-GFP foci using live-cell imaging of a Ch^16^-DSB-Tel strain encoding Rad52-GFP (TH6131) following DSB induction. *T* = 0 refers to time (min) when an elongated cell initiates cell division, that occurs ∼24 h following DSB induction, which includes the length of time taken for HO derepression following thiamine removal (∼16 h). (**B**) Live-cell imaging of Ch^16^-DSB-Tel *lacO* strain expressing *lacI-GFP* following DSB induction. The *LacO* array was integrated into the *dmf1* locus of Ch^16^-DSB-Tel, and the GFP-LacI-NLS was integrated into *dis1* (TH8734, TH8260). *T* = 0 refers to time (min) at which an elongated cell initiates cell division (see A). (**C**) Pedigree analysis of the third generation of cells carrying Ch^16^-DSB-Tel following DSB induction (TH6864). Colony pairs grown on YES and EMM-U plates are shown from three independent analyses (1–4,5–8,9–12). (**D**) PFGE analysis of genomic DNA from a wild type containing Ch^16^‐Tel-DSB (lane 1) and individual Ura^+^ colonies derived from daughter and grand-daughter cells from 3C (1–12). Sizes of Ch^16^-DSB-Tel (Ch^16^) and isochromosome (Ch^I^) are shown. Above is a schematic depicting the presence of a broken and resecting Ch^16^-DSB-Tel in each daughter cell (green sparks depicting Rad52-GFP foci), following DSB induction (red lightning) indicative of its replication by daughter and grand-daughter cells shown by PFGE. (**E**) Quantification of uninucleate cells positive for EdU incorporation depicting bulk DNA replication occurring normally in binucleate cells carrying Ch^16^-DSB-Tel (TH6864) at indicated times following thiamine removal to induce a DSB. SEM values are indicated. Scale bar = 10 μm.

To explore the fate of the unrepaired broken chromosomes following DNA damage checkpoint adaptation, a single cell microscopy time course was performed to visualise the broken unrepairable Ch^16^-DSB-Tel using Rad52-GFP (62). Following DSB induction, Rad52-GFP foci were observed in both daughter cells, consistent with unrepaired broken chromosomes being inherited by both daughters (Movie S1). Surprisingly, Rad52-GFP foci were also observed in both daughter cells for at least two generations following DSB induction (Figure 5A, 180–340 min and Supplementary Figure S6A–C). This suggested that the inherited broken minichromosomes were being replicated and segregated in daughter cells. Quantification of Rad52-GFP foci revealed that broken chromosomes were largely resolved during isochromosome formation over several generations. Consistent with this, 24 h following DSB induction >60% of Ch^16^-DSB-Tel cells carried Rad52-GFP foci, whereas at 48 h following DSB induction only 15% of Ch^16^-DSB-Tel cells were associated with Rad52-GFP foci (Supplementary Figure S6A–C).

To provide further evidence for replication of inherited broken minichromosomes, Ch^16^-DSB-Tel was visualised following integration of *lacO* repeat arrays into Ch^16^-DSB-Tel and expressing LacI-GFP that specifically binds to *lacO* arrays. Following break induction within Ch^16^-DSB-Tel, *lacO*/LacI-GFP foci were observed in daughter cells for several generations, consistent with the inherited broken chromosome being efficiently replicated and segregated in daughter cells (Figure 5B and Movie S2).

To further establish whether unrepaired broken chromosomes are replicated in daughter cells following DSB induction, individual elongated cells carrying Ch^16^-DSB-Tel encoding a *ura4* gene (Figure 5C) were segregated for two generations and allowed to form colonies on non-selective YES plates followed by replica plating onto EMM plates without uracil (EMM-Ura). We found all resulting daughter and granddaughter colonies were able to grow on EMM-Ura plates from three separate pedigree analyses (Figure 5C colonies 1–12), indicating the unrepaired broken minichromosome was inherited in daughter cells for two generations. These colonies on EMM-Ura plates also exhibited a range of colony sectoring patterns indicative of eventual loss of the *ura4* gene, Ch^16^ and CIN. PFGE analysis of all the resulting colonies derived from two generations of daughter cells from three independent experiments carried shorter derivatives of Ch^16^-DSB-Tel, consistent with isochromosome formation (Figure 5D). These findings indicate that an unrepaired broken chromosome is replicated by successive generations of daughter cells, while undergoing extensive end processing to form isochromosomes. Consistent with this, we further showed by successive pedigree analysis that the broken minichromosome was replicated for at least six generations (Supplementary Figure S6D).

To determine if the timing of endogenous DNA replication is impacted by the DNA damage checkpoint adaptation process, and whether the replication of the broken chromosome is coincident with bulk DNA replication, we examined EdU incorporation in cells carrying Ch^16^-DSB-Tel following DSB induction. In Ch^16^-DSB-Tel cells expressing *Drosophila melanogaster* deoxyribonucleoside kinase (DmdNK), together with the human equilibrative nucleoside transporter (hENT1) (*adh-Dm-dNK-adh-hENT1*), we found that EdU was not incorporated in elongated cells following DSB induction. In contrast, EdU incorporation was observed in 10–20% of the population by 30 h following DSB induction but was largely restricted to binucleate cells (G1/S phase), and normal length daughter and granddaughter cells following checkpoint adaptation (Figure 5E). These findings are concordant with the unrepaired broken minichromosome, Ch^16^-DSB-Tel, being replicated during normal S-phase and that endogenous DNA replication proceeds normally following adaptation to an unrepaired broken chromosome.

Following WGS analysis, the number of single nucleotide polymorphisms (SNPs) and indels detected on isochromosome was similar to the wild-type Ch^16^-DSB-Tel strain. These results together demonstrate that following checkpoint adaptation, daughter cells inherit an unrepaired broken chromosome that is subsequently faithfully replicated and segregated over multiple generations.

### Independently processed broken sister chromatids drive cell-to-cell heterogeneity and widespread CIN over multiple generations.

**Figure 6.**
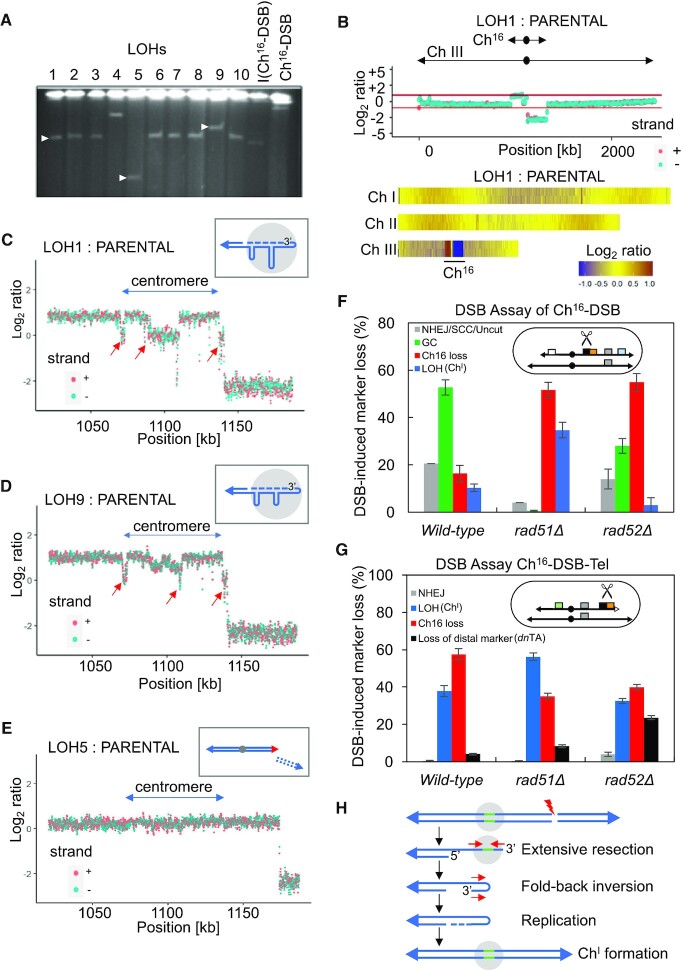
Break-induced isochromosomes associated with CIN arise from foldback inversions. (**A**) PFGE analysis of chromosomal DNA from individual wild-type Ura^+^ Ade^−^ G418^S^ (LOH) strains (lane 1–10) originating from Ch^16^-DSB, isolated after DSB induction. Distinct sizes of chromosomal elements LOH1(TH9245), LOH5 (TH9246) and LOH9 (TH9247) are indicated (white arrow). (**B**) Next generation sequence analysis of LOH strains. NGS analysis showing the log_2_ of the signal ratio between parental Ch^16^-DSB and LOH1 strain across Ch III. Locations of Ch^16^ and Ch III centromeres (circle) and telomeres (arrows) are indicated. Data acquisition and normalization were carried out as described in Materials and Methods. Heat map representation of NGS analysis of LOH1 showing three endogenous chromosomes. Yellow indicates a 1:1 ratio, red indicates signal intensity >1 and blue is <1. (**C**) NGS analysis of LOH1 across centromeric genome sequences. Red arrows point out non-duplicated spacer regions between deleted and duplicated segments exhibiting parental disomic copy number (log_2_ ratio of LOH: parental = 0). Schematic of LOH mechanism within centromeric region (grey). See text for details. (**D**) NGS analysis of LOH9 across centromeric genome sequences. Red arrows point out spacer regions, as indicated, and schematic as in (C). (**E**) NGS analysis of LOH5. Schematic of LOH mechanism by *de novo* telomere addition with new telomere (red), old telomeres (blue), and centromere (grey) indicated. (**F**) Percentage of DSB-induced marker loss in a wild-type (TH2125/6), *rad51Δ* (TH7356), or *rad52Δ* (TH2278), background carrying Ch^16^-DSB (Ch^16^‐RMGAH, illustrated). Levels of NHEJ/SCC/Uncut (Arg^+^ Hyg^R^ Ade^+^ His^+^); GC (Arg^+^ Hyg^S^ Ade^+^ His^+^); LOH (Arg^+^ Hyg^S^ Ade^−^ His^−^) predicted to arise from isochromosome formation (Ch^I^); and Ch^16^ loss (Arg^−^ Hyg^S^ Ade^−^ His^−^) are indicated. See also Supplementary Figure S1 for further details. S.E.M values are indicated. The data presented are from at least two independent biological repeats. (**G**) Percentage of DSB-induced marker loss in a wild-type (TH6874), *rad51Δ* (TH8075), or *rad52Δ* (TH8158), background carrying Ch^16^‐Tel-DSB, illustrated. Levels of NHEJ (Ura^+^ Ade^+^ G418^R^), LOH (Ura^+^ Ade^−^ G418^S^), Ch^16^ loss (Ura^−^ Ade^−^ G418^S^), and loss of distal marker (Ura^+^ Ade^+^ and G418^S^) are indicated, and predicted mechanisms of LOH shown (Ch^I^, isochromosome formation; *dn*TA, *de novo* telomere addition). S.E.M. values are indicated. The data presented are from at least two independent biological repeats. (**H**) Model for isochromosome (Ch^I^) formation resulting from extensive resection and foldback inversion. Telomeres (blue triangle), centromere (grey circle) inverted repeats (red arrows), replicated region (blue dashes) spacer regions (green) between deleted and duplicated segments exhibiting parental disomic copy number are shown. Adapted from (65).

We wished to determine the consequences of such post-adaptive DNA replication and end processing on genome stability in subsequent generations. We noted that colonies derived from single elongated cells carrying an unrepairable broken Ch^16^-DSB-Tel minichromosome in a wild-type background exhibited a unique series of colony sectoring patterns of *ura4* marker loss, indicative of CIN, when replica-plated from a non-selective YES plates to EMM-Ura plates (Figure 5C; Figure 2C). PFGE analysis revealed that CIN was associated with distinct minichromosome sizes in cells from individual LOH colonies (Figure 6A) or from individual cells within the same LOH colony (Supplementary Figure S7A), and is predominantly associated with isochromosome formation, confirming this chromosomal rearrangement to be a consequence of CIN. These findings further indicate that CIN arising from an unrepaired DSB leads to widespread heterogeneity.

A possible mechanism by which a broken chromosome could drive cell-to-cell heterogeneity is if broken sister chromatids arising from DNA replication are independently processed. To test this possibility pedigree analysis of DSB repair outcomes in daughter cells was performed. Following DSB induction in the parental cell, carrying the repairable minichromosome Ch^16^-DSB, revealed GC/GC (46.2%) GC/ (NHEJ or SCC) (0.8%); GC/LOH (12.0%); GC/Ch^16^ loss (6.7%); LOH/LOH (13.6%); LOH/Ch^16^ loss (11.0%); or Ch^16^ loss/ Ch^16^ loss (2.8%) daughter colony pairs (Supplementary Figure S7B). Thus, broken sister chromatids can be repaired independently. Similarly, DSB induction in the parental cell carrying the unrepairable minichromosome Ch^16^-DSB-Tel revealed LOH/LOH (55%), LOH/dead (19%) Ch^16^ loss/LOH (0.5%), dnTA/LOH (6%) dnTA/dnTA (2%) dead/dnTA (1%) and dead/dead (17%) daughter colony pairs (Supplementary Figure S7C). Thus, broken sister chromatids can be misrepaired independently. As broken sister chromatids can arise from replication of an inherited broken chromosome following adaptation, such independent misrepair of broken sister chromatids is likely to contribute to cell-to-cell heterogeneity after each successive division, and to widespread CIN.

### Sequence analysis of break-induced chromosomal rearrangements

To explore the possible mechanisms by which CIN leads to chromosomal rearrangements in more detail WGS analysis was performed on genomic DNA from LOH strains exhibiting different minichromosome sizes (LOH1, 5 and 9) shown by high resolution PFGE (Figure 6A). WGS analysis confirmed that the left arm of the minichromosome had been duplicated in LOH1 and 9 consistent with isochromosome formation when compared to that of the uncut parental strain carrying Ch^16^-DSB-Tel (Figure 6B, Supplementary Figure S8A and S8B). In contrast, the left arm of the minichromosome of LOH5 was not duplicated. Instead, the LOH5 minichromosome was found to have retained some of the right arm, and sequence analysis revealed that LOH had resulted from *de novo* telomere addition 158 kb centromere proximal from the *MATa* DSB site (46,54) (Supplementary Figure S8C).

Further sequence analysis of LOH1 and 9 suggested more complex genome rearrangements within the centromere (Figure 6C and D), in contrast to LOH5 (Figure 6E). A possible explanation for these rearrangements is if extensive end-processing facilitated the annealing of single-strand inverted repeat regions within the centromere to form a ‘fold-back’ looping structure. Following DNA replication, such structures result in chromosome arm duplication, as previously described (63,64), leading to isochromosome formation (21). A hallmark of fold-back inversions is the presence of non-duplicated ‘spacer’ regions between the inverted segments that display a parental disomic copy number (65) (Figure 6H). Consistent with this, NGS analysis revealed the presence of spacer regions (a Log_2_ ratio of 0) within the centromeric inverted repeats (*imr*, *dg*, *dh* and *irc*) exhibiting parental disomic copy number at the junction between deleted and duplicated segments of LOH1 and LOH9 (Figure 6C and D). Further, both LOH1 and LOH9 exhibit additional non-duplicated spacer regions within the centromere. We speculate that these regions have arisen through annealing of single-stranded inverted repeats that were subsequently not replicated following extension of the 3’ end during foldback inversion. This would result in their deletion from one side of the isochromosome and their appearance as non-duplicated regions (see Figure 6C and Figure 6D schematics).

Further genetic analysis indicated isochromosome formation to be independent of Rad51 but partially dependent on Rad52 (Figure 6F and G), consistent with a role for Rad52 in single-strand annealing of resected inverted repeats (66,67) thereby triggering chromosome arm duplication and isochromosome formation. Together, these findings support a model in which transgenerational extensive resection from a single-ended DSB through inverted repeats within the centromere leads to fold-back inversions, DNA replication and subsequently to isochromosome formation (Figure 6H) and to other chromosomal rearrangements.

## DISCUSSION

Impaired HR is implicated as a driver of CIN across a number of cancer types (9,10). Yet how impaired HR initiates and perpetuates CIN remains is poorly understood. In this study, using fission yeast as a model system, we establish a role for HR genes in suppressing break-induced CIN. Further, by following the fate of an unrepaired broken chromosome, we define an ordered series of molecular events through which failed HR drives widespread numerical and structural CIN.

Our findings reveal that a broken chromosome carrying a single-ended DSB, resulting from either failed HR repair or telomere loss, is subject to extensive end processing over multiple generations. We find that such transgenerational DSB end processing requires DNA damage checkpoint adaptation. We demonstrate that inherited broken chromosomes are subject to faithful DNA replication by successive daughter cells, thereby driving the propagation of CIN. We show that broken chromosomes are independently processed by successive daughter cells, leading to cell-to-cell heterogeneity. We determine that broken chromosomes arising from failed HR are either stabilized through spatially and temporally distinct misrepair mechanisms or are lost. Such misrepair mechanisms are independent of the major NHEJ or HR DSB repair pathways, and result in fold-back inversions or *de novo* telomere addition. These misrepair events predominantly lead to isochromosome formation in our model system but have the capacity to generate a spectrum of chromosomal rearrangements.

**Figure 7.**
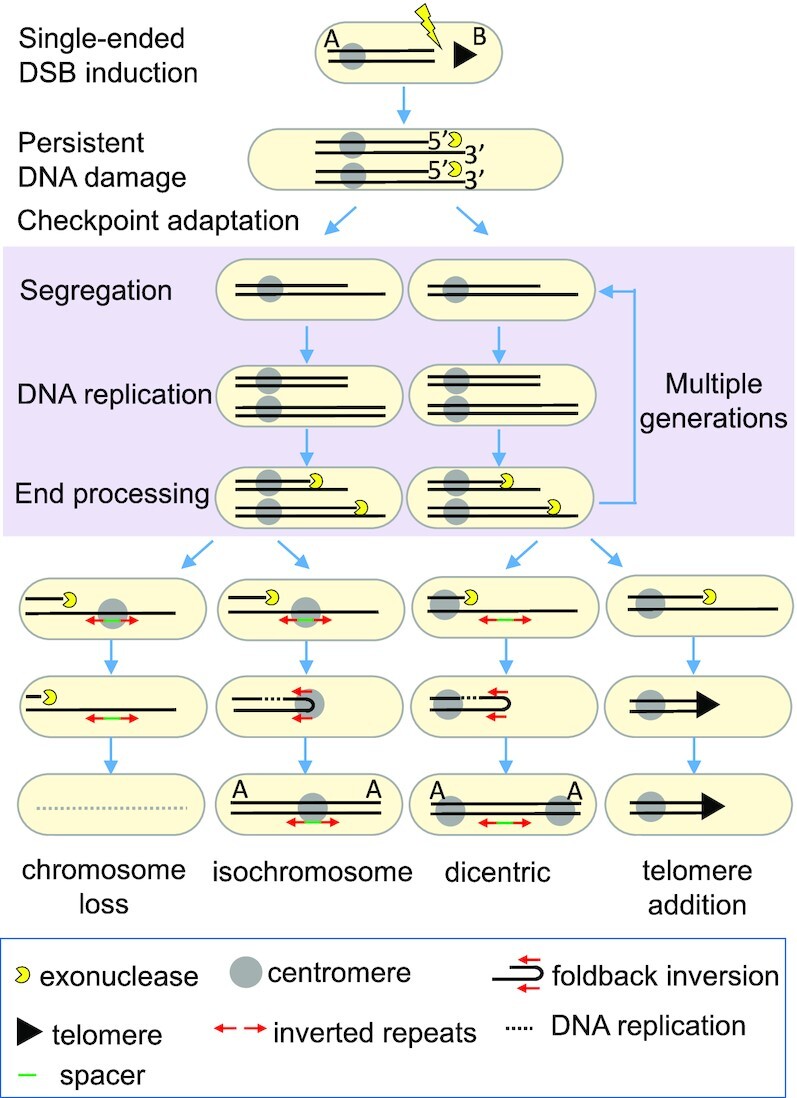
Model for break-induced chromosomal instability. A persistent single-ended DSB arising from impaired HR leads to post-adaptive cycles of segregation, DNA replication and transgenerational end processing (SERPent cycles; highlighted in purple), propagating widespread CIN. Observed and predicted outcomes of CIN are shown.

Together, these observations lead us to propose a new model for the initiation and perpetuation of CIN, in which an unrepaired single-ended DSB arising from failed HR, or telomere loss, gives rise to an unrepaired broken chromosome. This single-ended unrepaired broken chromosome is subject to post-adaptive cycles of segregation, DNA replication and DNA end processing (SERPent cycles). These cycles give rise to a population of daughter cells containing unrepaired broken chromosomes. SERPent cycles continue over multiple generations until unstable broken chromosomal elements are individually stabilized or lost within each of the progeny cells (Figure 7). This model explains how impaired HR drives CIN through facilitating extensive resection of a single-ended DSB that would normally be limited through completion of HR repair. Further, this model allows us to explain how a single-ended DSB associated with an unrepaired broken chromosome can lead to widespread chromosomal instability and to gross chromosomal rearrangements over multiple generations across the resultant population.

Our data support a single-ended DSB as a potent driver of CIN. Consistent with this we find that CIN, as determined by increased colony sectoring or increased levels of extensive LOH and Ch^16^ loss, is considerably elevated following DSB induction in HR mutants, or following telomere loss, both of which generate single-ended DSBs. Further, we find that isochromosome formation results from transgenerational resection of a single-ended DSB and is therefore a product of CIN. Such single-ended DSBs are predicted to arise from exposure to radiation and other clastogens, as well as through collapsed replication forks or telomere loss (68–70), suggesting such events may also drive CIN through this proposed mechanism. The identification of *S. pombe* orthologues of HR CIN genes as CIN genes in *S. cerevisiae* (57) (Supplementary Table S4), strongly suggests that the mechanism by which they drive CIN, described here, is evolutionarily conserved across all eukaryotes. These findings are consistent with and extend our mechanistic understanding of earlier observations in which HR genes have been found to suppress spontaneous and DSB-induced chromosomal rearrangements (21,71–74). We speculate that such chromosomal rearrangements may occur across multiple generations, which arise as a consequence of CIN, and are triggered by an unrepaired single-ended DSB.

We show that checkpoint adaptation facilitates CIN in *S. pombe*. These findings are consistent with reports of checkpoint adaptation contributing to genome instability in *S. cerevisiae* (36,38,75). We demonstrate that checkpoint adaptation requires degradation of checkpoint protein Chk1 via Btb3-Pcu3 that is activated during G2-M arrest. In mammalian cells, Chk1 degradation, through an Fbx6-containing E3 ligase, has been shown to terminate the DNA replication checkpoint (76), suggesting a conserved link between Chk1 degradation and checkpoint adaptation. Importantly, these findings in fission yeast indicate that break-induced CIN resulting from failed HR repair may be specifically targeted through blocking DNA damage checkpoint adaptation thereby preventing segregation and propagation of unrepaired broken chromosomes.

A key feature of such break-induced CIN is that following checkpoint adaptation, inherited broken chromosomes are faithfully replicated by daughter cells. This finding has considerable significance as this explains how unstable chromosomes are propagated across the population of daughter cells, thereby driving widespread cell-to-cell heterogeneity, rather than being otherwise limited to a single cell lineage. Further, replication of unstable intermediates coupled with independent processing of sister chromatids provides a mechanistic basis for rapid genetic variation, prior to chromosomal stabilization or loss. Our findings suggest that replication of the broken chromosome is accurate and consistent with bulk DNA replication. Thus, the post-adaptive DNA replication machinery, once initiated, appears unable to distinguish between an intact and a broken chromosome. These findings build on observations in which a broken chromosome was shown to be propagated in *S. cerevisiae* (23), suggesting post-adaptive replication of unrepaired broken chromosomes to be common across eukaryotes.

Our findings indicate that post-adaptive segregation, replication and transgenerational extensive processing of a single-ended DSB leads to a broad spectrum of chromosomal rearrangements across multiple generations. In this regard, we calculate that a single yeast cell carrying an unrepaired broken chromosome will have undergone up to ten SERPent cycles in the time taken to resect 200 kb from the break site to the centromeric inverted repeats, generating up to 1000 cells carrying unstable chromosomes. Crucially, each inherited and replicated sister chromatid is independently processed by daughter cells, and can thus undergo a number of possible fates including stabilization through partial or complete chromosomal arm duplication leading to telomere acquisition; stabilization through *de novo* telomere addition (46,54), or complete loss (21). Surprisingly, while break-induced replication (BIR) initiated from a one-ended DSB can maintain telomeres and trigger chromosomal rearrangements (77), BIR was a very minor contributor to chromosomal rearrangements in our Ch^16^-based assay despite the presence of a homologous ChIII template (46), or when a 3 kb region of ChI was integrated proximal to the break site in an effort to promote BIR using a subtelomeric region of ChI (Figure 3C and Supplementary Figure S3). Instead, our findings establish transgenerational Rqh1 and Exo1-dependent exonucleolytic activity as a major driver of broken chromosome catabolism and CIN.

Our data support a model in which extensive DNA 5' end resection, initiated through failed HR repair of a distal DSB, continues over hundreds of kilobases, and over multiple generations, until it resects through inverted repeats within the Ch^16^ centromere. Our findings suggest that annealing of these single-stranded inverted repeats promotes fold-back inversion, which following DNA replication, results in inverted chromosomal arm duplication, as previously described (37,64,78,79), and isochromosome formation (21). Similar observations were recently described for antifungal drug resistant clones associated with isochromosomes in *Candida albicans* (80). Importantly, our findings indicate that these chromosomal rearrangements are a consequence of CIN initiated by a single-ended DSB. However, fold-back inversions can potentially generate dicentric chromosomes and initiate breakage fusion bridge cycles, as previously described (63), and may further propagate CIN (Figure 7).

A feature of this study is that to harness the genetic potential of fission yeast to study CIN, we have focused on the fate of a single enzymatically broken experimentally derived nonessential minichromosome. We nevertheless anticipate that this model system may contribute to a broader understanding of CIN and how it is suppressed. It will be important to determine whether this mechanism is evolutionarily conserved and can lead to CIN in human cancers. In this respect, fold-back inversions are associated with genetic disease (65,81,82) and a variety of cancers (83–85). Further, fold-back inversions observed in human cancer cells, having escaped telomere-driven crisis, occur independently of classic DSB repair pathways (86,87), as is observed here. Moreover, as foldback inversions are initiated through extensive resection our finding that HR genes suppress CIN through preventing extensive resection suggests a new mechanism by which HR genes may function as tumour suppressors. Therefore, we anticipate that insights into the causes and propagation of CIN using this model system will contribute more broadly to the understanding of genetic disease and tumorigenesis.

## DATA AVAILABILITY

All materials and raw data are available upon request.

## Supplementary Material

gkad160_Supplemental_Files
