# Supplementary material for: Homologous recombination suppresses transgenerational DNA end resection and chromosomal instability in fission yeast
Source: Nucleic Acids Res. 2023 Mar 23;51(7):3205–22. doi: 10.1093/nar/gkad160 (PMC10123110; doi:10.1093/nar/gkad160)
Supplement: gkad160_Supplemental_Files [file gkad160_supplemental_files.zip › Supplementary Figs 14 02 23.pdf]

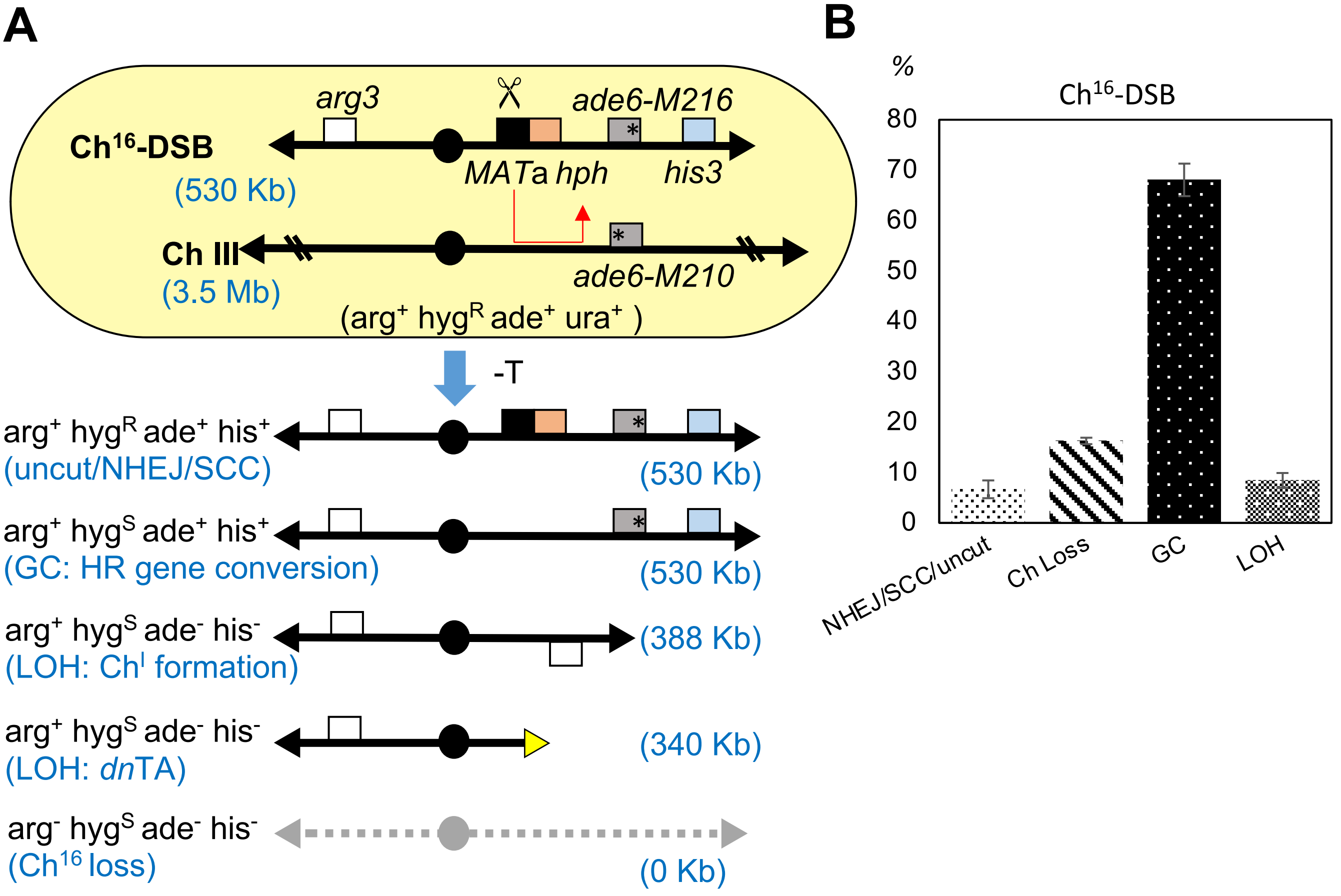

Fig. S1 DSB repair outcomes following a site specific DSB induction in repairable Ch^{16}-DSB (Ch16-RMYAH).(**A**) Schematic of Ch^{16}-DSB. Ch^{16}-DSB, ChIII, centromeric regions (black circles), complementary heteroalleles (*ade6-M216* and *ade6-M210*; grey with asterix denoting point mutations), and the *his3* marker (light blue) was inserted ~50kb downstream from *ade6-M216*, as previously shown (21). The *MATa* site (black) with an adjacent hygromycin resistance marker gene *hph* (salmon) was inserted into *spcc23B6.06* ~ 30kb upstream from *ade6-M216*. The *arg3* marker (white) was inserted into *spcc1795.09* on the left arm of the minichromosome. Derepression of pREP81X-HO on a plasmid (not shown) in the absence of thiamine (-T) generates a DSB uniquely at the *MATa* target site (scissors) after ~ 16h. Repair of HO-induced DSB by **NHEJ** results in retention of all markers resulting in an arg^{+} Hyg^{R} ade^{+} his^{+} phenotype as indicated. DSB repair by sister chromatid conversion (**SCC**) during S or G2 phase, in which one of the two sister chromatids is intact, and used as a repair template results in retention of all markers, resulting in an arg^{+} Hyg^{R} ade^{+} his^{+} phenotype, as indicated. This is indistinguishable from NHEJ in a wild-type background. DSB repair by interchromosomal gene conversion (**GC**) in which homologous ChIII is used as a repair template (red arrows) (53) results in loss of the *KanMX* gene adjacent to the *MATa* break site while the other markers are retained resulting in an arg^{+} Hyg^{S} ade^{+} his^{+} phenotype. Extensive loss of heterozygosity (**LOH**) in which genetic material centromere-distal to the break-site is lost results in an arg^{+} Hyg^{S} ade^{-} his^{-} phenotype, as indicated. LOH can result from cross-overs associated with gene conversion, break-induced replication, isochromosome formation, or *de novo* telomere addition (yellow triangle) (21). Failed DSB repair results in loss of the minichromosome (**Ch^{16} loss**), and loss of all of the markers, resulting in an arg^{-} Hyg^{S} ade^{-} his^{-} phenotype, as indicated. (**B**) Percentage of DSB-induced marker loss in wild-type cells containing Ch^{16}-DSB. The levels of non-homologous end joining/sister chromatid conversion/uncut (NHEJ/SCC/uncut), gene conversion (GC), minichromosome loss (Ch^{16} loss), and LOH are shown. s.e.m. values are indicated. The data presented are from at least two independent biological repeats. For further experimental details see (44).

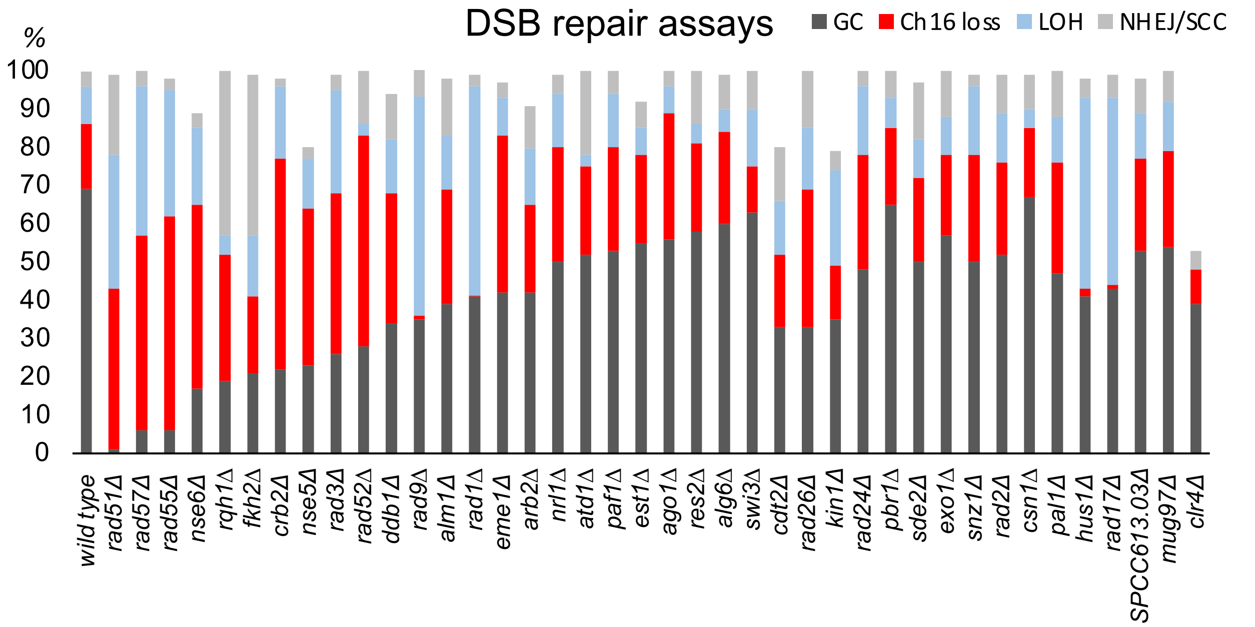

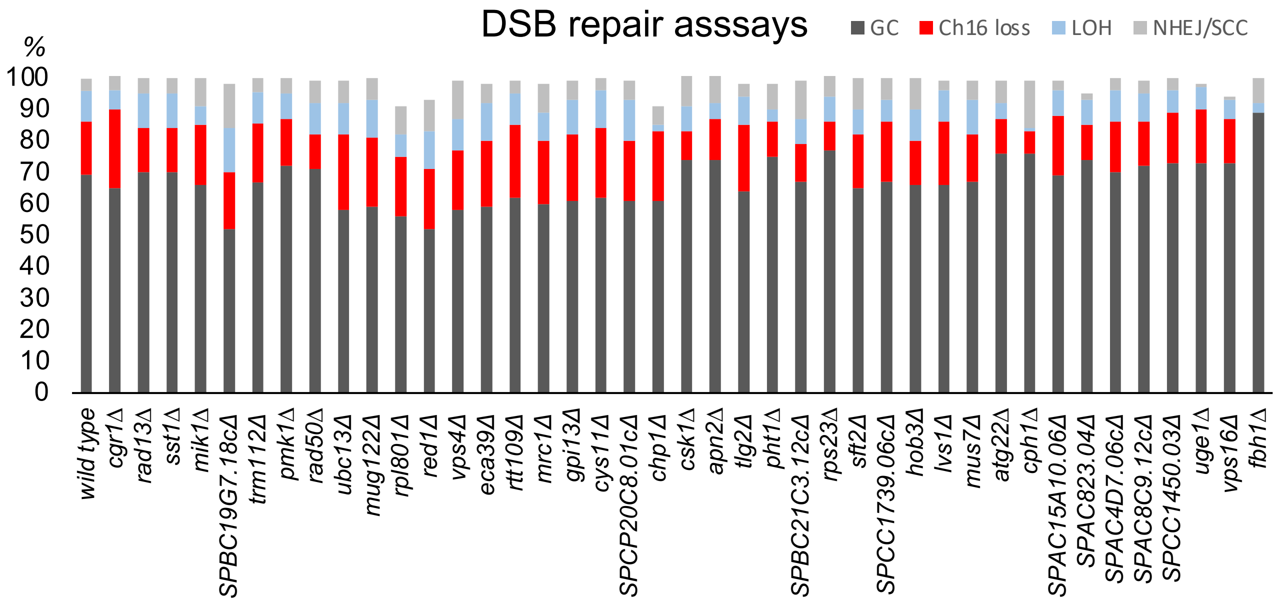

Fig. S2 DNA repair profiles of 81 sectoring mutants. Sectoring mutants were crossed into Ch[16]-RMYAH and DSB assays performed as described in Materials and Methods. Tabulated values are given in Table S3. Where outcomes total <100% of wild-type levels reflects spontaneous Ch16 loss.

A

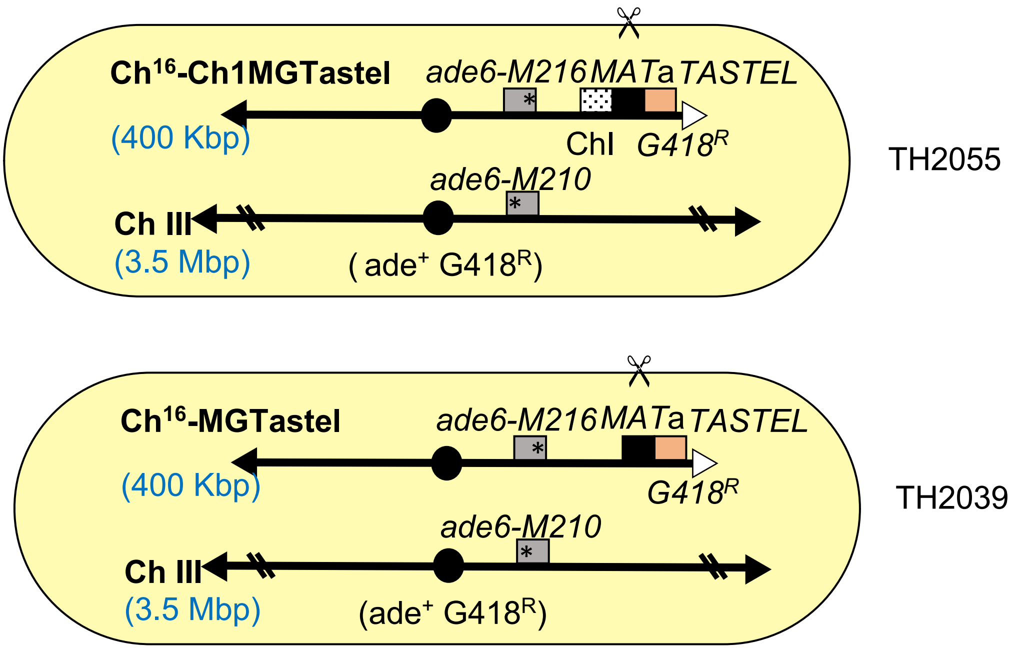

B

Genetic Background	Strain Number	Colonies Scored	Spontaneous Ch^{16} loss % *ade*$^-$ G418^S	NHEJ/Uucut% *ade*$^+$ G418^R	Loss of distal markers % *ade*$^+$ G418^S	Failure to Repair % *ade*$^-$G418^S	
Wild type	TH2055	2052	3.6 ± 0.4	4.9 ± 0.5	3.8 ± 0.6	87.7 ± 0.2	ChI-tastel
Wild type	TH2039	1820	3.5 ± 0.2	24.2 ± 1.7	3.4 ± 0.9	68.9 ± 2.4	tastel

Fig. S3 Genetic analyses of minichromosome repair after DSB induction in TH2055 and TH2039 (**A**) Ch^{16}-Ch1MGTastel was constructed to test whether by introducing a short region of homology (3Kb) to a sub-telomeric sequence on chromosome I (ChI) situated 79 kb from the telomere increased levels of Break-Induced Replication, predicted to result in increased levels of Ade$^+$ G418^S cells. Instead, this resulted in increased failed repair, and is therefore used to structurally disrupt HR in this study. This 3 kb of homology to ChI (black dots) was engineered at the centromere-proximal side of the *MATa* site (black box) to form Ch^{16}-Ch1MGTastel in TH2055. Ch^{16}-Ch1MGTastel in TH2055 was constructed in a similar manner as Ch^{16}-MGTastel in TH2039. The ChI/*MATa*/G418^R/TASTEL DNA fragments were excised from pTTHF01 via EagI-KpnI digests and integrated at the same site as the *MATa*/G418^R/TASTEL fragments in TH2039, i.e. the *SPCC132.03* locus. The relative locations of the *ade6* (grey box) and *KanMX6* genes (salmon) within the minichromosomes are shown. (**B**) Genetic analyses of minichromosome repair after DSB induction in TH2055 and TH2039. Values are mean ± standard error from three independent experiments using different isolates.

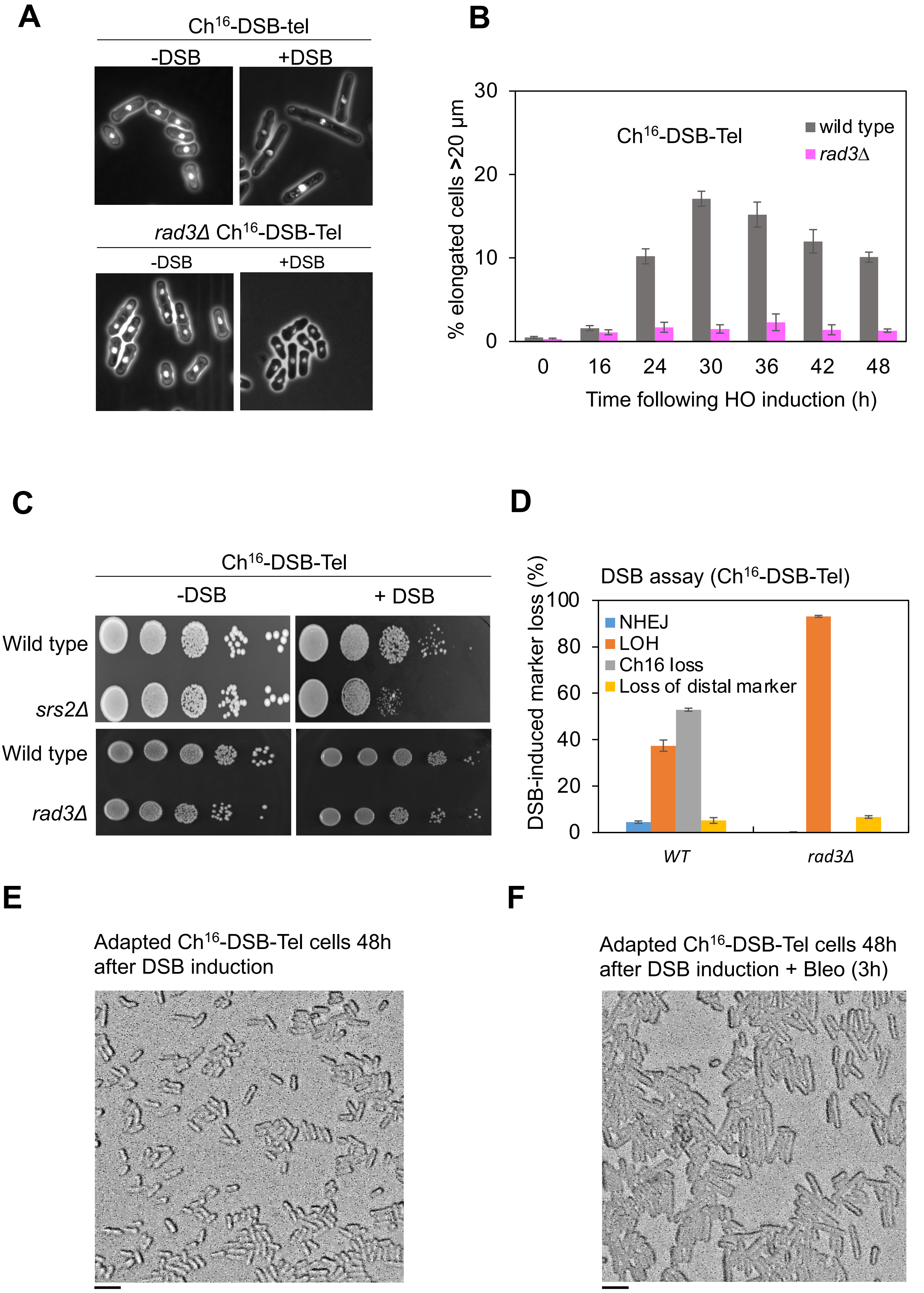
A
Ch16-DSB-tel
-DSB
+DSB
rad3Δ Ch16-DSB-Tel
-DSB
+DSB
B
Ch16-DSB-Tel
wild type
rad3Δ
% elongated cells >20 µm
30
20
10
0
0
16
24
30
36
42
48
Time following HO induction (h)
C
Ch16-DSB-Tel
-DSB
+ DSB
Wild type
srs2Δ
Wild type
rad3Δ
D
DSB assay (Ch16-DSB-Tel)
DSB-induced marker loss (%)
100
80
60
40
20
0
NHEJ
LOH
Ch16 loss
Loss of distal marker
WT
rad3Δ
E
Adapted Ch16-DSB-Tel cells 48h after DSB induction
F
Adapted Ch16-DSB-Tel cells 48h after DSB induction + Bleo (3h)

Fig. S4 Cell division is facilitated by DNA damage checkpoint adaptation. (**A**) Cell morphology analysis of wild-type Ch[16]-DSB-Tel (TH6864) *or rad3*Δ Ch[16]-DSB-Tel (TH9233) cells following DSB induction. Prior to DSB induction (-DSB), all cells are normal length (12 μm). Upon DSB induction (+DSB), Ch[16]-DSB-Tel cells undergo cell cycle arrest, resulting in an elongated phenotype (>20 μm) after 20h. In contract, *rad3*Δ Ch[16]-DSB-Tel cells do not show elongated phenotypes. Size bar = 10μm (**B**) Percentage of cell population with elongated phenotype (>20 μm) at time points indicated following *pREP81x-HO* derepression following thiamine removal (+DSB) in wild-type or *rad3*Δ cells carrying Ch[16]-DSB-Tel. (**C**) Serial dilution of wild-type Ch[16]-Tel-DSB (TH6864), *srs2*Δ Ch[16]-DSB-Tel (TH9519) and *rad3*Δ Ch[16]-DSB-Tel (TH9233) strains in the presence or absence of thiamine. Plates were incubated at 32°C for 3 days. (**D**) DSB analysis in wild-type and *rad3Δ* backgrounds carrying Ch[16]-DSB-Tel. Data represents mean ± s.e.m. (**E**) Challenging checkpoint adapted cells with DNA damage agents. Adapted Ch[16]-DSB-Tel cells following 48h DSB induction. (**F**) Adapted cells were treated with Bleocin (0.4 μg/ml) for 3h in the absence of thiamine. Size bar = 10μm

Pai et al. Figure S5

A

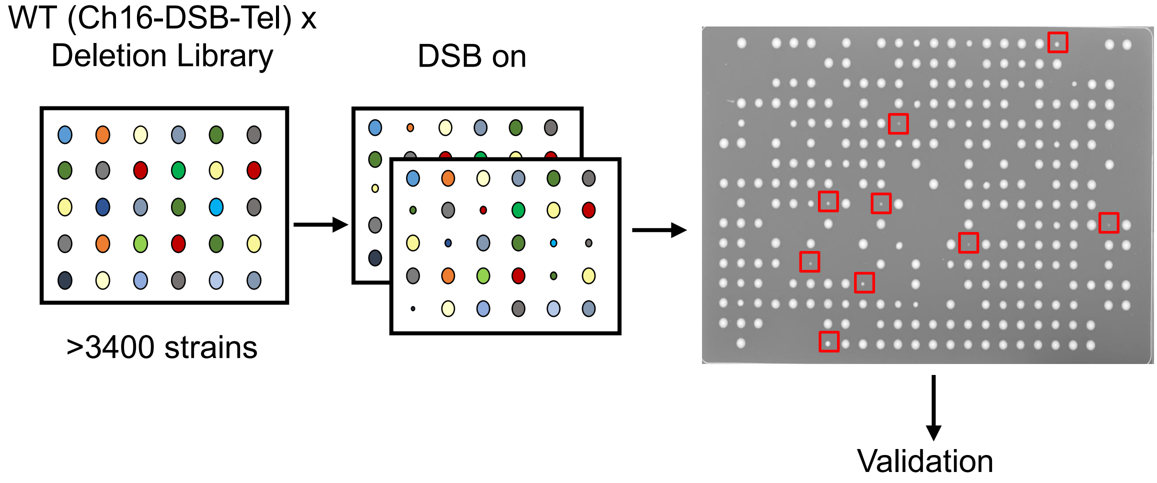

Fig.S5 Screen for DNA damage checkpoint adaptation mutants. The Bioneer *S. pombe* haploid deletion libraries were screened for synthetic lethality (SL) with Ch[16]-DSB-Tel following DSB induction. Mutants were identified as smaller or absent colonies on the selection plates without thiamine after 48-72 hours of incubation at 32°C. Red squares indicate mutants with a reduction in colony size on the thiamine-free plate.

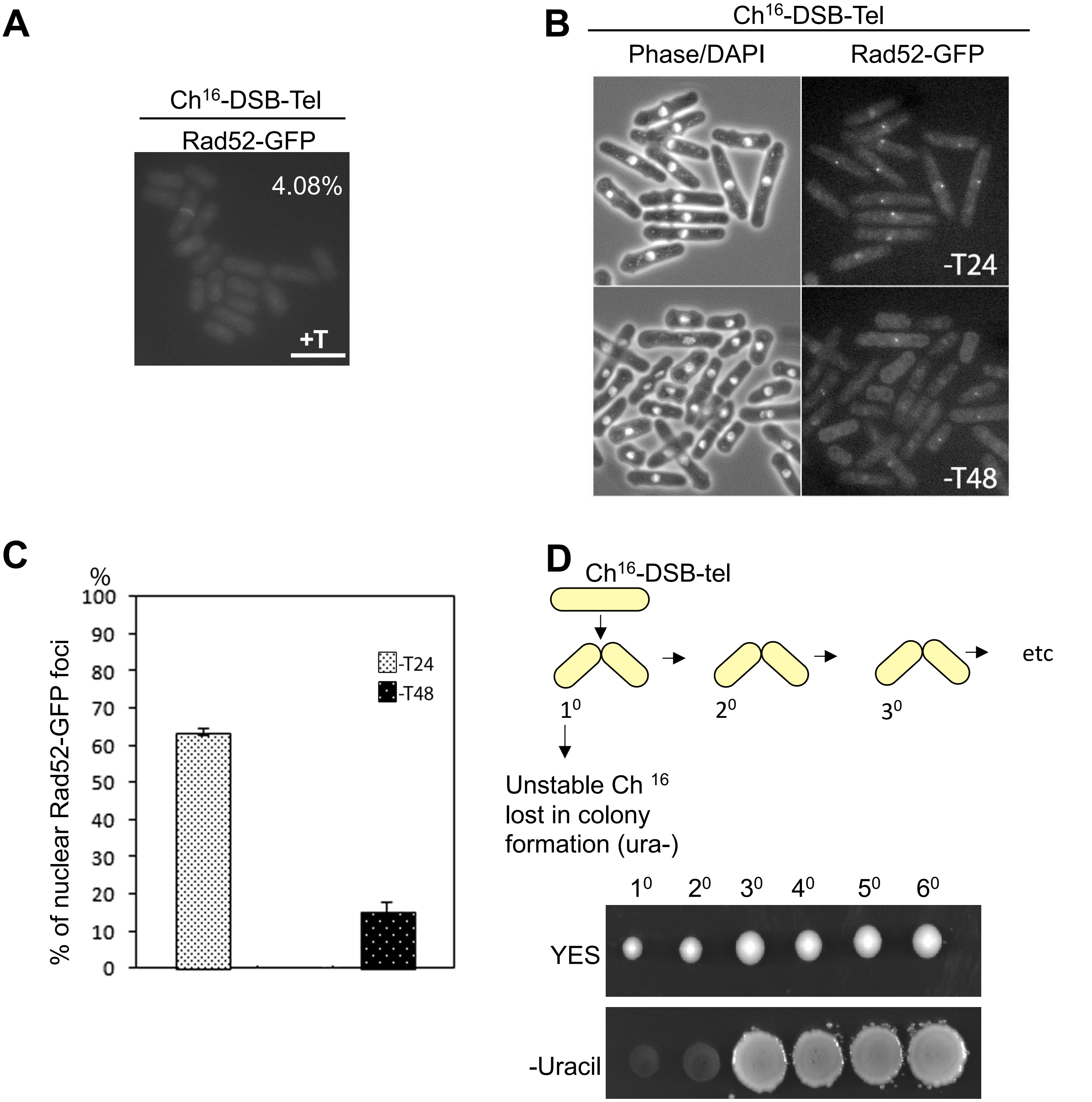

Fig. S6 Analysis of Rad52 foci in Ch^{16}-DSB-Tel cells following DSB induction. (**A**) Microscopic analysis of Rad52-GFP foci in Ch^{16}-DSB-Tel cells (TH6131) in the absence of DSB induction; + T indicates in the present of 5 µg/ml thiamine. Scale bar =10 µm. (**B**) Analysis of Rad52-GFP foci in Ch^{16}-DSB-Tel cells at 24 hrs and 48 hrs following DSB induction. -T indicates in the absence of thiamine. (**C**) Quantification of Rad52-GFP foci in Ch^{16}-DSB-Tel cells following DSB at indicated time points in B. Data are the mean of two experiments and error bars (±s.e.) are shown. (**D**) Representative pedigree of 6 sequential daughters from a single elongated Ch^{16}-DSB-Tel cell following DSB induction. 1° signifies the colony arising from the first daughter cell, 2° signifies the colony arsing from the second, 3° signifies the colony arising from the third, 4° signifies the colony arising from the fourth daughter cell, 5° signifies the colony arising from the fifth daughter cell, and 6° signifies the colony arising from the sixth daughter cell. These sequential daughter cells were then replica plated onto Uracil- plates to show the presence or absence of minichromosomes.

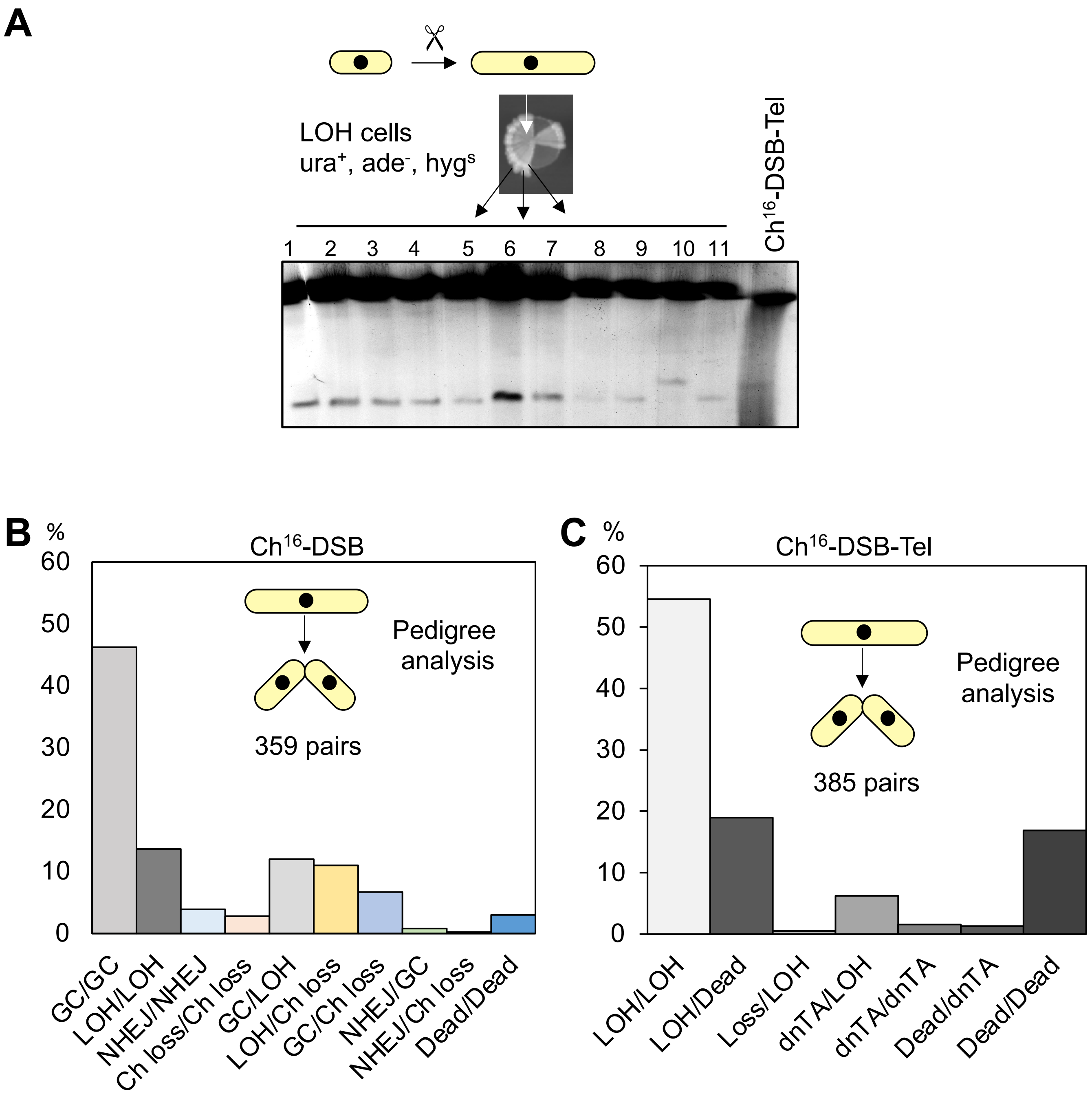

Fig. S7 An unresolved single-ended DSB drives cell-to-cell heterogeneity and CIN. **(A)** PFGE analysis of chromosomal DNA from wild type Ch^{16}-DSB-Tel descendants (1-11) derived from a single elongated Ch^{16}-DSB-Tel cell following DSB induction. (**B**) Broken sister chromatids are repaired or resolved independently. DSB repair outcomes were analysed in paired daughter cells following a site specific DSB induction in Ch^{16}-DSB (TH2125). (**C**) Pedigree analysis of paired daughter cells following a site specific DSB induction in Ch^{16}-DSB-Tel (TH6864). Resultant colonies were replica plated and scored for growth on selective plates to identify phenotypes depicted in Figure 1C or cell death.

A

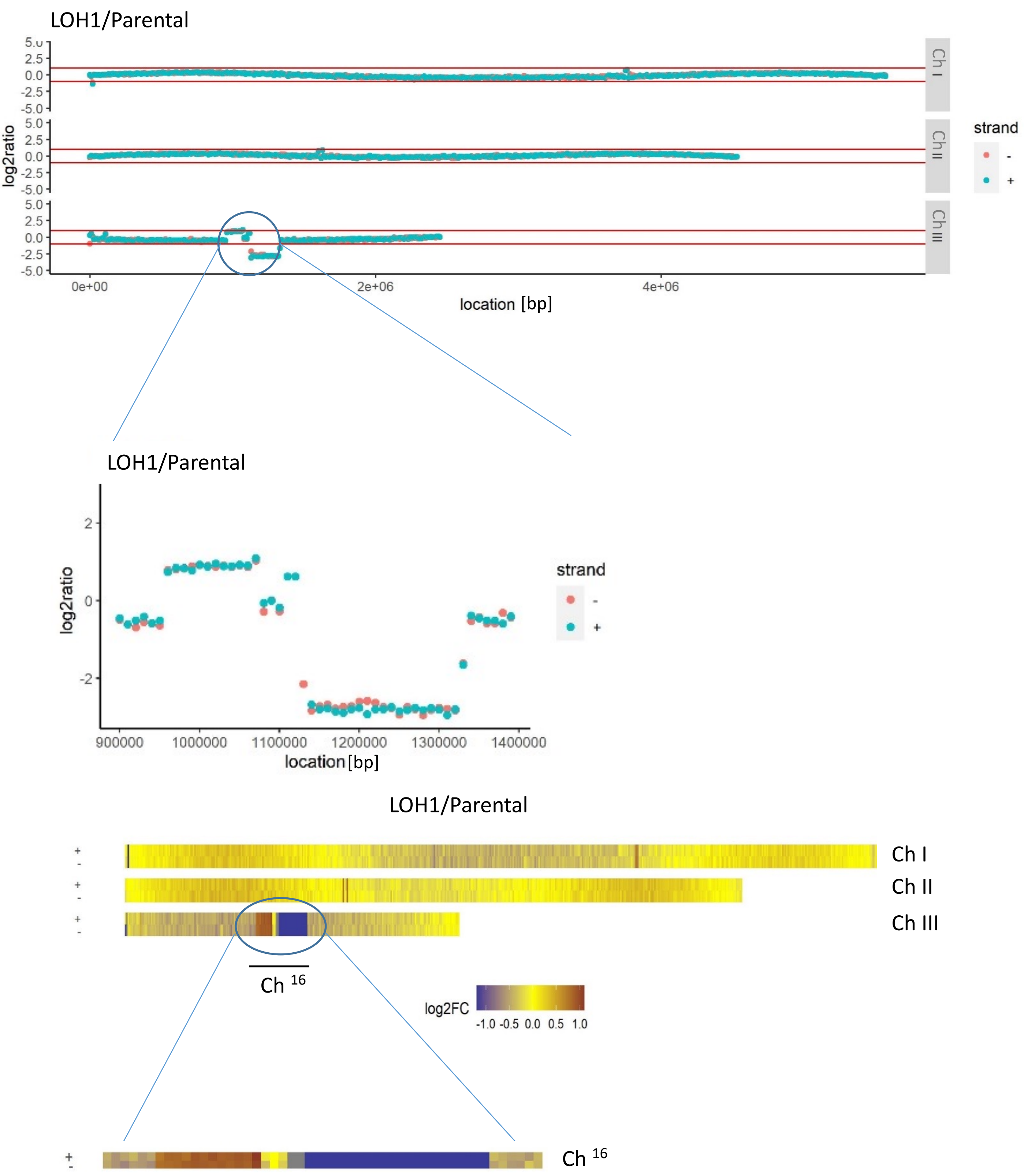

LOH1/Parental
log2ratio
5.0
2.5
0.0
-2.5
-5.0
Ch I
Ch II
Ch III
strand
-
+
0e+00
2e+06
4e+06
location [bp]
LOH1/Parental
log2ratio
2
0
-2
900000
1000000
1100000
1200000
1300000
1400000
location [bp]
strand
-
+
LOH1/Parental
Ch I
Ch II
Ch III
Ch 16
log2FC
-1.0 -0.5 0.0 0.5 1.0
Ch 16

B

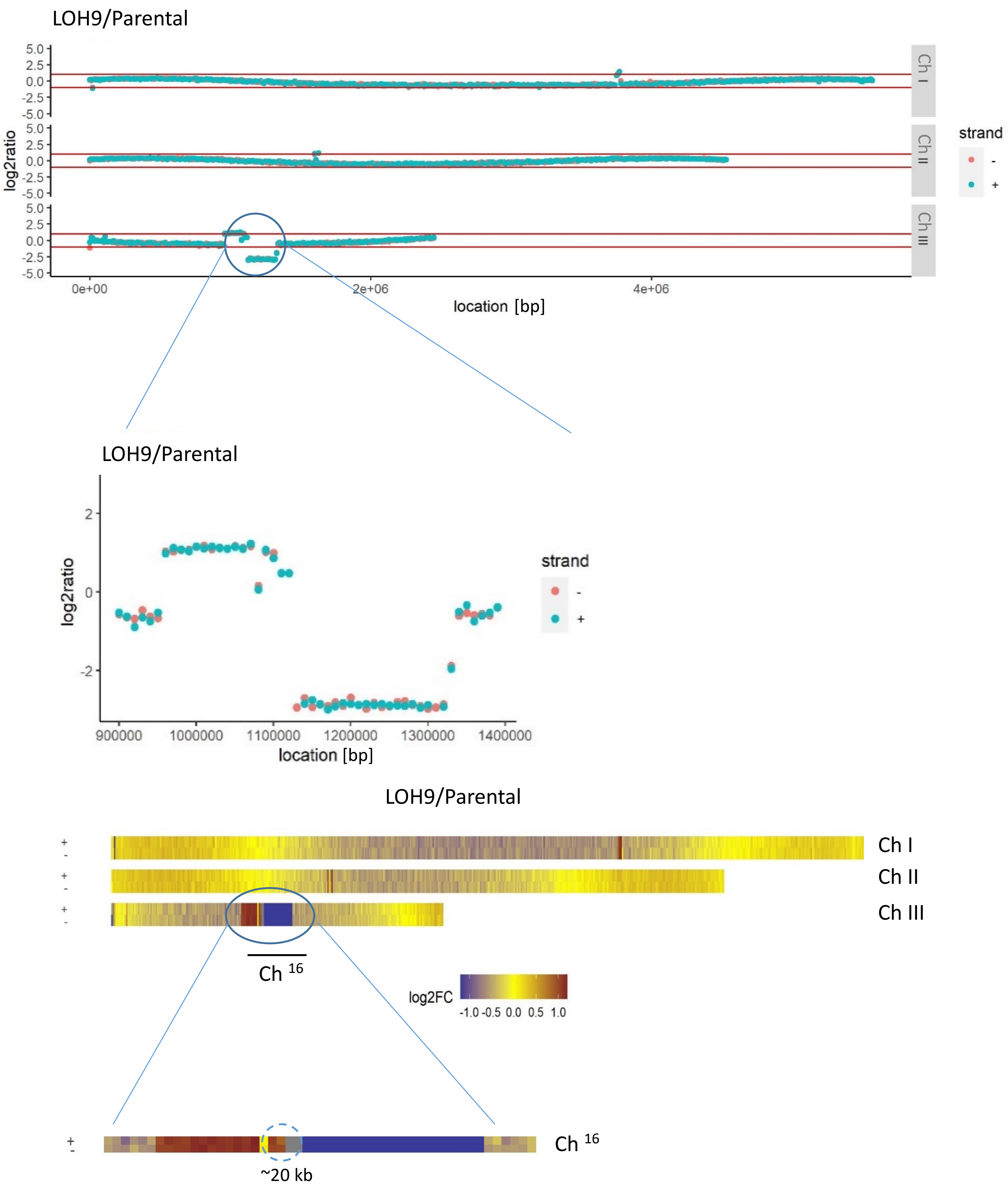

LOH9/Parental
log2ratio
5.0
2.5
0.0
-2.5
-5.0
Ch I
Ch II
Ch III
strand
-
+
0e+00
2e+06
4e+06
location [bp]
LOH9/Parental
log2ratio
2
0
-2
900000
1000000
1100000
1200000
1300000
1400000
location [bp]
strand
-
+
LOH9/Parental
+
-
Ch I
Ch II
Ch III
Ch 16
log2FC
-1.0 -0.5 0.0 0.5 1.0
Ch 16
~20 kb

C

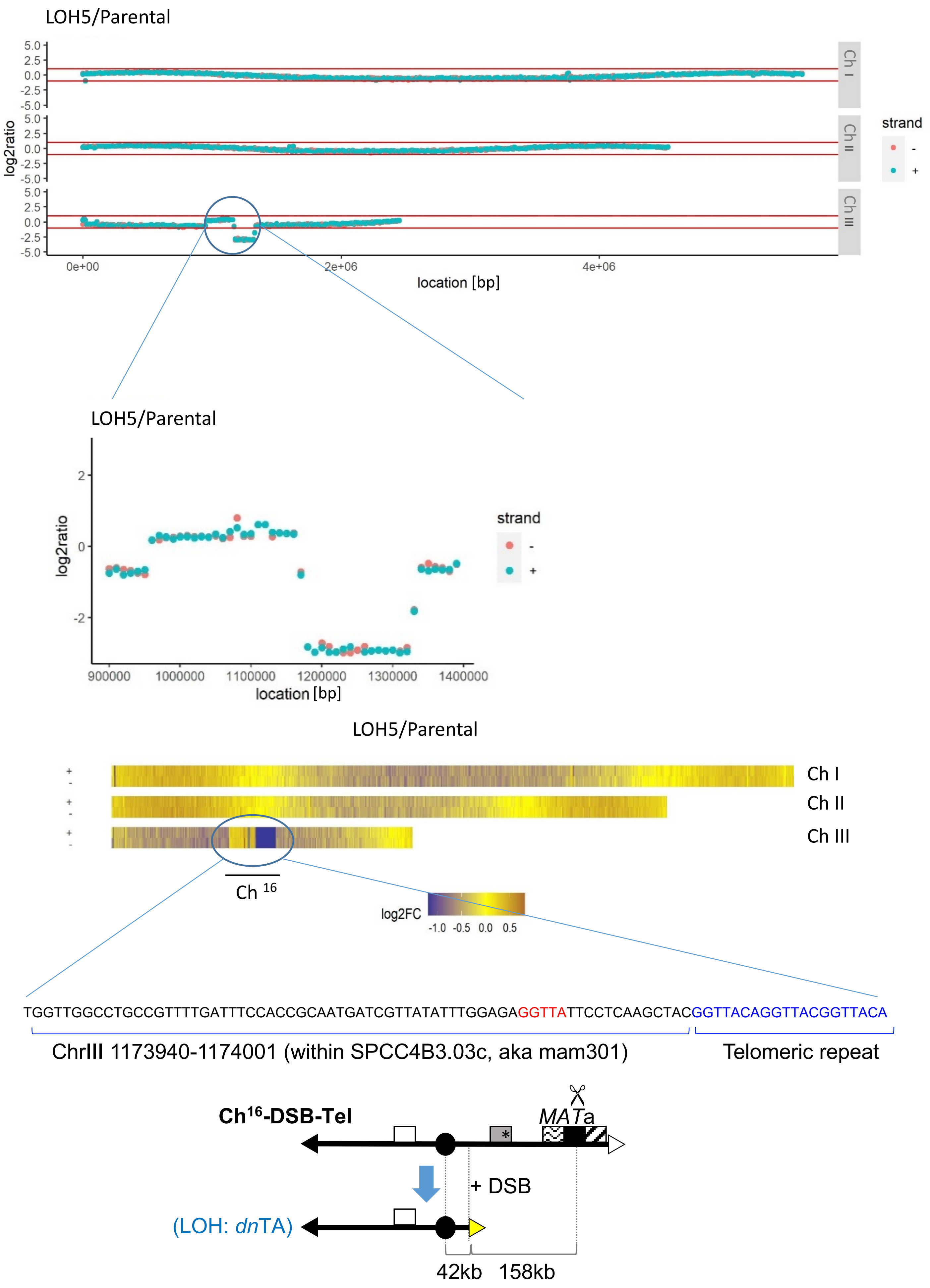
LOH5/Parental
log2ratio
5.0
2.5
0.0
-2.5
-5.0
Ch I
Ch II
Ch III
strand
-
+
0e+00
2e+06
4e+06
location [bp]
LOH5/Parental
log2ratio
2
0
-2
900000
1000000
1100000
1200000
1300000
1400000
location [bp]
strand
-
+
LOH5/Parental
Ch I
Ch II
Ch III
Ch 16
log2FC
-1.0 -0.5 0.0 0.5
TGGTTGGCCTGCCGTTTTGATTTCCACCGCAATGATCGTTATATTTGGAGAGGTTATTCCTCAAGCTACGGTTACAGGTTACGGTTACA
ChrIII 1173940-1174001 (within SPCC4B3.03c, aka mam301)
Telomeric repeat
Ch16-DSB-Tel
MATa
*
+ DSB
(LOH: dnTA)
42kb
158kb

Fig. S8 A single DSB can lead to a spectrum of chromosomal rearrangements over generations. (**A**) WGS analysis of LOH1 in (**Figure 6C**). WGS analysis showing the log_2 of the signal ratio between parental Ch^{16}-DSB-Tel and LOH1 strain (TH9245). Data acquisition and normalization were carried out as described in Materials and Methods. LOH1/Parental density heat-map displayed on three endogenous chromosomes. Yellow indicates a 1:1 ratio, the red is higher than 1 and blue is lower than 1 (**B**) WGS analysis was carried out for LOH9 (TH9247) compared to parental Ch^{16}-DSB-Tel. (**C**) WGS analysis was carried out for LOH5 (TH9246) compared to parental Ch^{16}-DSB-Tel. Sequence of *de novo* telomere addition site and its position within Ch16-DSB-Tel are indicated. A possible seed sequence for de novo telomere addition is indicated in red. See also Fig. 2
